# Comparative analysis of 6-lead and single-lead consumer-grade electrocardiograms: diagnostic accuracy, numerical agreement, and inter-rater reliability

**DOI:** 10.1093/ehjdh/ztag086

**Published:** 2026-06-11

**Authors:** Myoung Jung Kim, Sujeong Eom, Juwon Kim, Seung-Jung Park, Kyoung-Min Park, Young Keun On, Sunghoon Joo, Mineok Chang, Yeongyeon Na, Minje Park, Jeonghwa Lim, Taehyung Yu, Hyun Jin Ahn, Ju Youn Kim

**Affiliations:** Division of Cardiology, Department of Internal Medicine, Seoul St Mary's Hospital, College of Medicine, The Catholic University of Korea, Seoul, Republic of Korea; Biosignal R&D Team, VUNO Inc., Seoul, Republic of Korea; Division of Cardiology, Department of Internal Medicine, Heart Vascular Stroke Institute, Samsung Medical Center, Sungkyunkwan University School of Medicine, 81 Irwon-ro, Gangnam-gu, Seoul 06351, Republic of Korea; Division of Cardiology, Department of Internal Medicine, Heart Vascular Stroke Institute, Samsung Medical Center, Sungkyunkwan University School of Medicine, 81 Irwon-ro, Gangnam-gu, Seoul 06351, Republic of Korea; Division of Cardiology, Department of Internal Medicine, Heart Vascular Stroke Institute, Samsung Medical Center, Sungkyunkwan University School of Medicine, 81 Irwon-ro, Gangnam-gu, Seoul 06351, Republic of Korea; Division of Cardiology, Department of Internal Medicine, Heart Vascular Stroke Institute, Samsung Medical Center, Sungkyunkwan University School of Medicine, 81 Irwon-ro, Gangnam-gu, Seoul 06351, Republic of Korea; Biosignal R&D Team, VUNO Inc., Seoul, Republic of Korea; Biosignal R&D Team, VUNO Inc., Seoul, Republic of Korea; Biosignal R&D Team, VUNO Inc., Seoul, Republic of Korea; Biosignal R&D Team, VUNO Inc., Seoul, Republic of Korea; Biosignal R&D Team, VUNO Inc., Seoul, Republic of Korea; Biosignal R&D Team, VUNO Inc., Seoul, Republic of Korea; Biosignal R&D Team, VUNO Inc., Seoul, Republic of Korea; Division of Cardiology, Department of Internal Medicine, Heart Vascular Stroke Institute, Samsung Medical Center, Sungkyunkwan University School of Medicine, 81 Irwon-ro, Gangnam-gu, Seoul 06351, Republic of Korea

**Keywords:** Wearable ECG devices, 6-lead electrocardiogram, Single-lead electrocardiogram, Diagnostic accuracy

## Abstract

**Aims:**

Consumer-grade electrocardiogram (ECG) devices enable accessible rhythm monitoring. Recently developed six-lead handheld ECGs promise improved signal quality and diagnostic performance; however, comparative validation remains limited. Using 12-lead ECGs as reference, we compared diagnostic performance and quantitative agreement of a 6-lead and single-lead consumer ECGs.

**Methods and results:**

In this prospective single-centre study, 194 arrhythmia-clinic patients (498 paired recordings) underwent simultaneous 10-s 12-lead and 30-s 6-lead ECG acquisition, followed by sequential 30-s single-lead recording. Two blinded electrophysiologists interpreted ECGs. Diagnostic performance for predefined rhythm categories (sinus rhythm, atrial/ventricular premature complex, atrial fibrillation/flutter/tachycardia, atrioventricular block, others) was assessed by sensitivity, specificity, and accuracy. Waveform agreement was evaluated using Bland–Altman analysis and intraclass correlation coefficients (ICC) accounting for repeated measures. Sensitivity was higher for the 6-lead ECG for ectopic beats, atrial flutter, and first-degree atrioventricular block, with comparable specificity across categories. Overall diagnostic accuracy was 98.6% (95% confidence interval, 97.4–99.6) for the 6-lead and 96.9% (94.7–98.7) for the single-lead ECG. Agreement with the 12-lead ECG was higher for the 6-lead device for PR interval (ICC 0.89; 0.85–0.92) and QRS amplitude (0.96; 0.94–0.97) than for the single-lead device (0.57; 0.48–0.63 and 0.04; −0.13–0.21, respectively). Bland–Altman analysis demonstrated generally narrower limits of agreement for the 6-lead ECG. Findings were consistent in patient-level sensitivity analyses.

**Conclusion:**

The 6-lead handheld ECG demonstrated a higher point estimate for diagnostic accuracy and closer agreement with the 12-lead ECG than the single-lead smartwatch ECG, supporting its use for arrhythmia assessment and interval measurement.

## Graphical Abstract

**Graphical Abstract:**
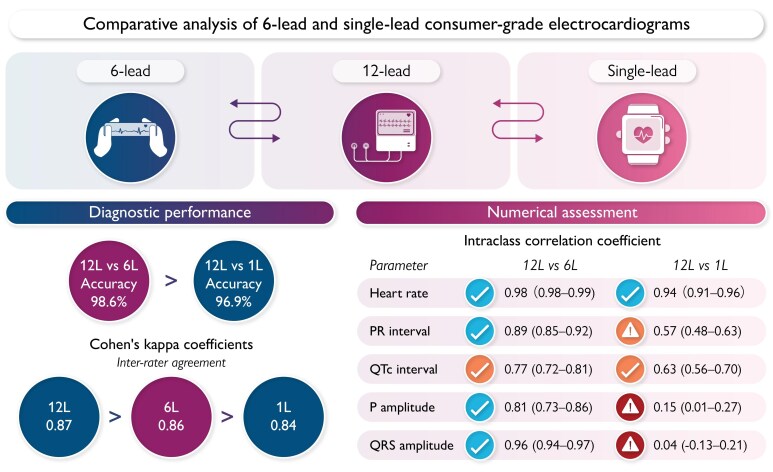

## Introduction

Consumer-grade electrocardiogram (ECG) devices have become essential tools in digital health and cardiology. They enable real-time cardiac rhythm monitoring outside of traditional clinical settings. Photoplethysmography-based smartwatches demonstrated the initial feasibility of atrial fibrillation (AF) detection; and the subsequent development of AF detection algorithms in single-lead ECG devices enabled clinical diagnosis.^1,2^ Beyond AF detection, wearable devices have shown clinical utility in identifying various arrhythmias, including tachy- and brady-arrhythmias in ambulatory settings.^3–5^ Moreover, deep learning algorithms applied to single-lead ECGs have demonstrated cardiologist-level performance in arrhythmia classification.^6^ However, despite these advantages, single-lead ECGs have inherent limitations, including low spatial resolution, limited P-wave visibility, and variable signal quality.^7^ To overcome these limitations, devices capable of recording six limb leads have emerged, potentially offering greater value without sacrificing convenience. This expanded lead set enables more precise rhythm classification^8^ and enhanced measurement of intervals, including the QT interval.^9,10^

However, comparative evaluations of single-lead and 6-lead consumer ECGs are limited, often omitting rigorous statistical analyses, such as Bland–Altman plots, intraclass correlation coefficients, and Cohen’s κ. This limits the interpretation of waveform agreement and inter-rater variability, highlighting the need for a systematic head-to-head evaluation. To address this, we conducted a head-to-head evaluation of single-lead and six-lead consumer ECGs against a 12-lead standard, assessing their diagnostic performance, waveform interval and amplitude agreement, and inter-rater reliability among expert readers in a real-world setting.

## Methods

### Study design and participants

This prospective, single-centre observational study was conducted at the Samsung Medical Center, a tertiary medical centre in Republic of Korea, from September 2023 to August 2024. The primary objective was to evaluate the diagnostic concordance and signal parameter accuracy of consumer-grade ECG devices, specifically a 6-lead handheld ECG (HATIV® P30, VUNO Inc., Seoul, Republic of Korea; hereinafter HATIV), and a single-lead smartwatch-based ECG (Galaxy Watch 5, Samsung Electronics, Suwon, Republic of Korea; hereinafter Galaxy), against the conventional 12-lead ECG system (Philips TC70 Cardiograph PageWriter, Philips, Amsterdam, Netherlands; hereinafter Philips), which served as the clinical gold standard.

Eligible participants were patients aged ≥19 years visiting the cardiac arrhythmia clinic. To reflect real-world use and to allow for repeat paired recordings, we preferentially enrolled patients with a routine follow-up appointment scheduled within approximately 3 months. All participants provided written informed consent and demonstrated the ability to comply with study instructions. Exclusion criteria included physical disability preventing the use of the 6-lead handheld ECG device; the presence of an implanted intracardiac device, such as pacemaker, implantable cardioverter-defibrillator, cardiac resynchronization therapy device, or ventricular assist device; or any condition deemed unsuitable by the investigator's clinical judgment.

### Ethics approval and consent

The Institutional Review Board of Samsung Medical Center (Seoul, Republic of Korea) approved this study (approval number: SMC 2022-12-112-011), and all participants provided written informed consent before enrolment. The study was conducted in accordance with the principles of the Declaration of Helsinki.

### Data collection

Baseline characteristics were systematically collected from all the participants, including demographic information, vital signs (blood pressure and heart rate), anthropometric measurements (body mass index and body surface area), and relevant comorbidities. Medication history within 90 days was recorded. Additionally, available laboratory results (blood tests) and echocardiographic findings were collected to supplement the baseline clinical data.

### Electrocardiogram acquisition

**Figure 1:**
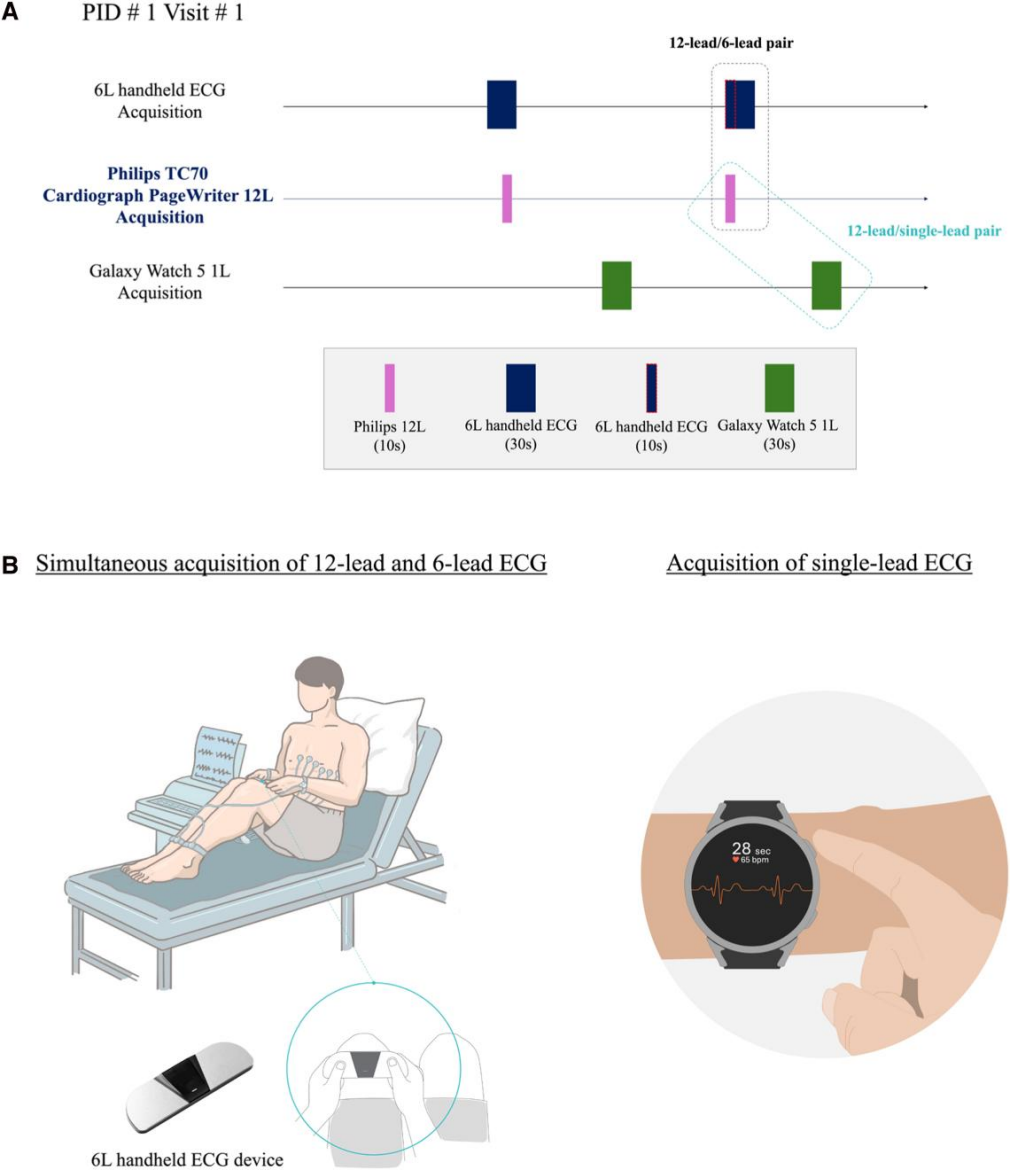
Illustration of ECG acquisition and mapping for each visit. (*A*) Although multiple ECG pairs were acquired during each visit and all collected ECGs were annotated, only the last ECG pair from each patient visit was used for the primary analyses in this manuscript (as indicated by the dotted boxes). The 12-lead and 6-lead ECGs were acquired simultaneously, followed by the recording of a single-lead ECG. To ensure precise temporal alignment, only the 10-s segment of the 6-lead ECG that was synchronized with the 12-lead ECG (red dotted box) was used for analysis. In contrast, the full 30-s recording of the single-lead ECG (green box) was included in the analysis. (*B*) This figure illustrates the procedure used for ECG acquisition in this study. On the left, a subject is seated for the simultaneous acquisition of 12-lead (Philips TC70 Cardiograph PageWriter, Philips, Amsterdam, Netherlands) and 6-lead (HATIV® P30, VUNO Inc., Seoul, Republic of Korea) ECGs, enabling temporal alignment between the two recordings. On the right, a subject is shown recording a single-lead ECG using a consumer-grade smartwatch (Galaxy Watch 5, Samsung Electronics, Suwon, Republic of Korea). COPYRIGHT VUNO Inc. ECG, electrocardiogram.

All ECG recordings were acquired by a single medical professional (M.J.K.), who was proficient in obtaining standard 12-lead ECGs, handheld 6-lead ECGs, and smartwatch-based single-lead ECGs. He had no financial or professional relationship with the manufacturer of the devices. As depicted in *Figure 1A*, 12-lead and 6-lead ECGs were acquired simultaneously, followed by collection of the single-lead ECG. Although multiple ECG pairs were generated per visit; and all were annotation, only the last ECG pair from each visit was utilized. Subsequent ECGs were collected during follow-up visits. To ensure precise alignment, only the 10-s segment of the 6-lead ECG that was temporally synchronized with the 12-lead recording was selected for analysis. As the single-lead ECG was acquired sequentially, the entire 30-s recording was utilized.

A standard 10-s 12-lead ECG was obtained using a Philips system with meticulous electrode placement following the American Heart Association guidelines.^11^ A 30-s 6-lead ECG was recorded using the HATIV. This device uses three contact points to derive six frontal-plane leads (I, II, III, aVR, aVL, and aVF). Specifically, the embedded electrodes for both thumbs provided contacts corresponding to the left and right arms, while a third contact at the left thigh approximated the left leg electrode. To facilitate simultaneous data collection, the participants remained seated during acquisition. Following this, a 30-s single-lead ECG (lead I) was recorded using the Galaxy. This was achieved by having the participant place a finger of the opposite hand onto the watch's dedicated electrode (button), while the watch is worn on the wrist, thereby establishing a closed circuit for lead I acquisition (*Figure 1B*).

The 12-lead and single-lead ECG systems sampled signals at a rate of 500 Hz, a widely accepted resolution for clinical-grade electrocardiographic interpretation. In contrast, the 6-lead ECGs were recorded at a sampling rate of 250 Hz. Although this rate is typically considered adequate for rhythm analysis and interval measurements, the lower sampling rate may result in reduced sensitivity to high-frequency components.

### Expert annotation

To prevent bias, two board-certified electrophysiologists independently annotated all the ECGs in a blinded manner. The annotation process involved both rhythm interpretation and manual beat labelling.

For rhythm interpretation, each ECG recording was assigned to one of the predefined rhythm categories: sinus rhythm, atrial premature complex (APC), ventricular premature complex (VPC), atrial fibrillation (AF), atrial flutter (AFL), atrial tachycardia (AT), atrioventricular (AV) block, and others (rhythms not falling into the above categories). Rhythms were defined according to the standard ECG diagnostic criteria. 12-lead/single-lead pairs were excluded from the diagnostic performance assessment if a patient's rhythm appeared to have changed between the time the 12-lead ECG was obtained and when the single-lead ECG was performed. If two expert readers had discrepant rhythm interpretations, a consensus was reached through discussion.

**Figure 2:**
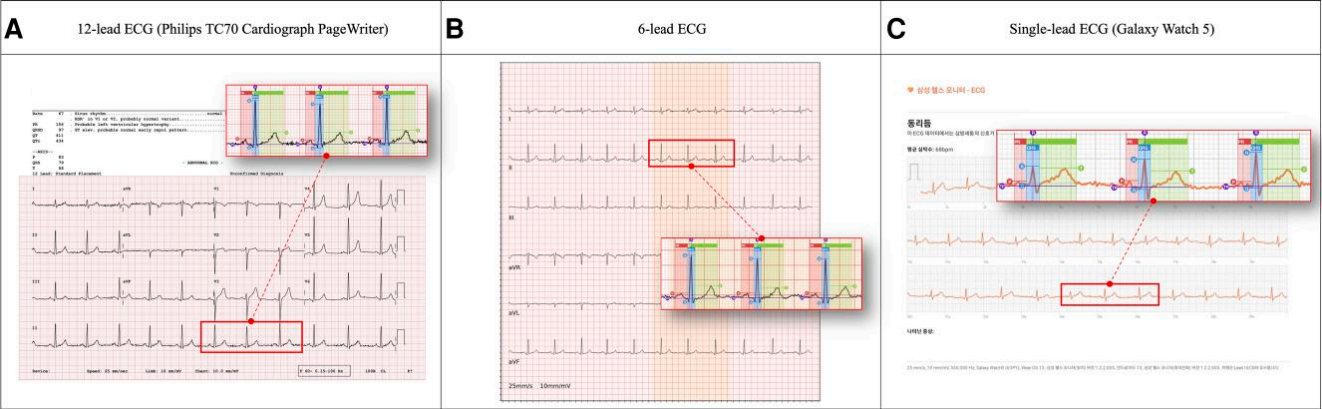
Representative examples of ECG papers and annotated ECG beats for the extraction of numerical parameters. All ECGs shown are from a single patient at a single time point, recorded sequentially using three different devices. The rhythm was interpreted as sinus rhythm. The annotation process included rhythm interpretation and manual labelling of fiducial points—P-wave onset, QRS onset/offset, T-wave offset, and P/R/T peaks—on three representative, consecutive, and noise-free beats. For the 12-lead and 6-lead ECGs, lead II was used as the primary reference lead for beat labelling, while lead I was used for the single-lead ECG. The following ECG parameters were extracted from the labelled beats: RR interval (ms), PR interval (ms), QRS duration (ms), QT interval (ms), heart rate-corrected QT (QTc, ms) using Bazett’s formula, and the amplitudes (mV) of the P-wave, QRS complex, and T-wave. ECG, electrocardiogram.

The following ECG parameters were measured from the labelled beats: RR interval (ms), PR interval (ms), QRS duration (ms), QT interval (ms), heart rate-corrected QT (QTc) interval (ms) by Bazett’s formula, and amplitudes of the P-wave, QRS complex, and T-wave (mV). Lead II served as the reference lead for beat labelling and amplitude measurements in the 12-lead and 6-lead ECGs, while the single-lead ECG corresponded to lead I (*Figure 2*). Heart rate (beats per minute, bpm) was derived from RR intervals. For the temporally aligned recordings, beats were identified on the 12-lead ECG and matched to the corresponding cardiac cycles on the 6-lead ECG to ensure beat-to-beat comparison within the same synchronized 10-s segment. For each numerical ECG parameter, such as PR interval and QRS duration, the average across three consecutive representative beats was calculated to serve as a representative value for each individual ECG recording. Subsequently, the representative values from each of the two expert readers were averaged to establish the final representative value for each ECG recording, which was then used for all subsequent analyses. Specific exclusions were applied during the parameter calculation. ECGs were excluded from the PR interval and P-wave amplitude calculations if a P-wave was absent in all labelled beats, such as due to AF. Additionally, if non-consecutive beats were selected for labelling, the ECGs were excluded from the heart rate and QTc interval analyses. In addition to expert interpretation, automated diagnostic outputs provided by the 6-lead and single-lead ECGs were collected for each ECG recording.

### Statistical analysis

The clinical characteristics were summarized using descriptive statistics. Continuous variables are presented as the mean with standard deviation (SD), while categorical variables are presented as numbers with percentages.

To account for repeated measurements from the same subject, we implemented a unified non-parametric cluster bootstrapping approach (1000 iterations resampled at the patient level).^12^

For rhythm interpretation, the 12-lead ECGs were used as reference standards for rhythm interpretation. The diagnostic performances of the 6-lead ECG and single-lead ECG were primarily evaluated using sensitivity and specificity for each rhythm category, with the overall accuracy reported as a complementary metric. Ungradable recordings were classified as incorrect in the primary analysis (intention-to-diagnose). The 95% confidence intervals (CI) were estimated using the participant-level bootstrapping method described previously. To compare the overall accuracies of the devices, we performed a paired comparison using McNemar’s exact test on the intersection set of recordings with valid classifications for both devices. As repeated recordings were obtained from the same participants, we additionally confirmed this comparison in a patient-level sensitivity analysis using one recording per participant. Interrater agreement for rhythm interpretation by the two readers across 12-lead, 6-lead, and single-lead ECGs was assessed using Cohen's kappa coefficient, with 95% CI estimated via cluster bootstrapping.^13^ The diagnostic performance of the automated interpretations from both devices was assessed against expert annotations. This study was considered exploratory. Representative ECG reports from the 6-lead and single-lead ECGs are shown in Supplementary material online, *Figure S1*.

Device agreement for numerical ECG parameters was rigorously assessed using two analytical methods. Paired comparisons between 12-lead/6-lead and 12-lead/single-lead ECGs were conducted using the Bland–Altman method.^14^ Bland–Altman analyses were performed using a random-intercept mixed-effect model for paired differences to account for within-participant correlation. We report the mean difference (bias) and 95% limits of agreement (LOA), with the LOA derived from the estimated variance components as the expected range containing 95% individual-level differences. A narrower LOA indicates more consistent measurements, whereas the mean difference reflects a systematic offset, with a value of zero indicating no systematic bias between the two measurement methods. All differences were calculated as 12-lead values minus 6-lead or single-lead values. To evaluate inter-device reliability for numerical parameters, an intraclass correlation coefficient (ICC) for absolute agreement (ICC(2,1)), with 95% CI estimated via cluster bootstrapping, was calculated.^15^ Furthermore, as the Bland–Altman method takes raw differences with positive or negative values as an input, the absolute differences between devices were assessed by their mean (SD).

For a sensitivity analysis addressing repeated measures, we repeated the above analyses using one recording per participant acquired during the first visit (patient-level analysis) (see Supplementary Material  *S1*). As an additional sensitivity analysis, the diagnostic assessment was repeated after excluding ungradable recordings (see Supplementary Material  *S2*).

Subgroup analyses were performed as exploratory analyses to investigate device performance under varying clinical conditions (see Supplementary Material  *S3*).

All statistical analyses were performed using Python (v3.11) with the SciPy and Statsmodels libraries. Statistical significance was defined as a two-sided *P*-value of < 0.05.

## Results

### Study population

Two hundred patients were prospectively enrolled with a median visit count three times and a median visit interval of 119 days (from the enrolment date to the first follow-up visit). After excluding six participants owing to withdrawal of consent and ECG data omission, 498 ECGs from 194 patients remained eligible.

The mean age of the study population was 56.7 years, and 70.6% were men. Baseline AF was present in 140 patients (72.2%), predominantly paroxysmal (111 patients), and in fewer persistent cases (26 patients). Other arrhythmias included AFL in 34 patients (17.5%), AT in 32 (16.5%), APC in 67 (34.5%), and VPC in 38 (19.6%) patients. Cardiovascular comorbidities were also present, including hypertension in 73 patients (37.6%), heart failure in 21 patients (10.8%), and valvular heart disease in 12 patients (6.2%) (*Table 1*).

**Table 1:** Baseline characteristics of study population

Demographics	Participants (*n* = 194)
**Male**	137 (70.6)
**Age**	56.7 ± 13.9
**BMI (kg/m^2^)**	25.0 ± 3.6
**BSA (m^2^)**	1.8 ± 0.2
**SBP (mmHg)**	125.8 ± 18.1
**DBP (mmHg)**	71.3 ± 12.4
**HR (bpm)**	78.4 ± 13.6
**Comorbidities**	
**Hypertension**	73 (37.6)
**Diabetes mellitus**	28 (14.4)
**Heart failure**	21 (10.8)
**Cerebrovascular accident**	10 (5.2)
**Peripheral artery disease**	1 (0.5)
**Myocardial infarction**	4 (2.1)
**Coronary artery bypass graft**	1 (0.5)
**Atrial fibrillation** ^a^	140 (72.2)
** Paroxysmal**	111
** Persistent**	26
** Permanent**	0
**Atrial flutter**	34 (17.5)
**Atrial tachycardia**	32 (16.5)
**Atrial premature complexes**	67 (34.5)
**Ventricular premature complexes**	38 (19.6)
**Sick sinus syndrome**	2 (1.0)
**Atrioventricular block**	11 (5.7)
**Left bundle branch block**	2 (1.0)
**Right bundle branch block**	6 (3.1)
**Valvular heart disease**	12 (6.2)
**Valve operation history**	10 (5.2)
**Medication history**	
**Anti-hypertensive**	45 (23.2)
**B-blocker**	46 (23.7)
**Flecainide**	20 (10.3)
**Propafenone**	41 (21.1)
**Pilsicainide**	0 (0.0)
**Dronedarone**	20 (10.3)
**Amiodarone**	7 (3.6)
**Sotalol**	1 (0.5)
**Echocardiography**	
**LVEF (%)**	62.3 ± 5.3
**E (m/s)**	0.7 ± 0.3
**A (m/s)**	0.7 ± 0.2
**e’ (m/s)**	0.1 ± 0.0
**E/e’**	9.1 ± 4.9
**LA diameter (mm)**	39.7 ± 6.9
**LAVI (mL/m^2^)**	36.2 ± 14.0
**Blood test**	
**BUN (mg/dL)**	15.2 ± 3.9
**Cr (mg/dL)**	0.9 ± 0.2
**eGFR (mL/min/1.73 m2)**	89.0 ± 16.7
**NT-proBNP (pg/mL)**	241.4 ± 456.1
**Na (mmol/L)**	140.4 ± 2.5
**K (mmol/L)**	4.4 ± 0.4

Continuous variables are presented as the mean with standard deviation (SD), while categorical variables are presented as numbers with percentages. Echocardiographic and blood test summaries were calculated using available data, as these results were not available for all participants.

^a^Atrial fibrillation (AF) subtype data were missing for 3 of 140 patients with AF; therefore, subtype counts do not sum to 140.

BMI, body mass index; BSA, body surface area; SBP, systolic blood pressure; DBP, diastolic blood pressure; HR, heart rate; LVEF, left ventricular ejection fraction; LA diameter, left atrial diameter; LAVI, left atrial volume index; BUN, blood urea nitrogen; Cr, serum creatinine; eGFR, estimated glomerular filtration rate; NT-proBNP, *n*-terminal pro–B-type natriuretic peptide; Na, serum sodium; K, serum potassium

### Diagnostic performance of consumer-grade ECG devices

**Figure 3:**
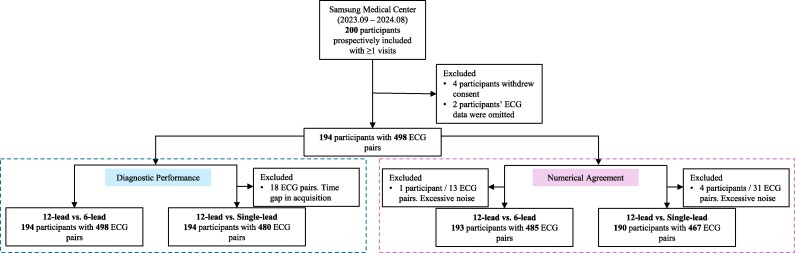
Study population and data flowchart. ECG, electrocardiogram.

Eighteen 12-lead/single-lead pairs (3.75%) were excluded from the rhythm interpretation analysis because of rhythm differences due to the acquisition time gap (*Figure 3*). A detailed summary of rhythm discordance attributable to acquisition time gap is provided in Supplementary material online, *Table S1* and Supplementary material online, *Figure S2*. The sensitivity and specificity according to the rhythm category are summarized in *Table 2*. The 6-lead ECG showed higher sensitivity than the single-lead ECG for ectopic beats (APC and VPC), atrial flutter (AFL), and atrioventricular block (AV block), whereas the specificity was generally high and comparable between devices across rhythm categories. All three AV block cases were first-degree AV block. Overall diagnostic accuracy was 98.6% (95% CI, 97.4–99.6) for the 6-lead ECG and 96.9% (94.7–98.7) for the single-lead ECG. In a paired comparison restricted to recordings with valid classifications for both devices (*n* = 480), McNemar’s test did not show a statistically significant difference in overall accuracy [6-lead: 98.5% (97.0–99.4); *P* = 0.0963]. No 6-lead cases were ungradable (0.0%), whereas five single-lead recordings were ungradable (1.04%); these were considered incorrect in the primary analysis. Sinus rhythm was the most prevalent, followed by AF. Both 6-lead and single-lead ECGs have demonstrated considerable robustness in AF detection. The Cohen’s Kappa coefficients for inter-rater agreement between the two electrophysiologists in each device were 0.87 (95% CI, 0.79–0.94) (12-lead), 0.86 (0.77–0.94) (6-lead), and 0.84 (0.77–0.90) (single-lead). The agreement between the two raters in rhythm interpretation was highest in the 12-lead, followed by the 6-lead and single-lead. In the patient-level sensitivity analysis, the results were consistent with those of the main analysis for overall diagnostic performance and inter-rater agreement, with the same ordering across modalities (see Supplementary Material  *S1* and Supplementary material online, *Table S2*). Similarly, the sensitivity analysis excluding ungradable recordings yielded results consistent with the primary analysis, except that the sensitivity for VPC became comparable between the two devices (see Supplementary Material  *S2* and Supplementary material online, *Table S3*).

**Table 2:** Diagnostic performance of consumer-grade ECG devices compared to standard 12-lead ECG

12L vs. 6L						
498 pairs. 194 participants						
	*n*	Sensitivity (95% CI)	Specificity (95% CI)	Accuracy (95% CI)	PPV (95% CI)	NPV (95% CI)
**Sinus rhythm**	425	99.5 (98.8–100.0)	93.2 (85.5–98.5)	98.6 (97.4–99.6)	98.8 (97.7–99.8)	97.1 (93.1–100.0)
**APC**	23	87.0 (68.2–100.0)	100.0 (100.0–100.0)	99.4 (98.6–100.0)	100.0 (100.0–100.0)	99.4 (98.5–100.0)
**VPC**	9	100.0 (100.0–100.0)	100.0 (100.0–100.0)	100.0 (100.0–100.0)	100.0 (100.0–100.0)	100.0 (100.0–100.0)
**AF**	35	97.1 (89.7–100.0)	99.8 (99.3–100.0)	99.6 (99.0–100.0)	97.1 (91.7–100.0)	99.8 (99.2–100.0)
**AFL**	1	100.0 (0.0–100.0)	100.0 (100.0–100.0)	100.0 (100.0–100.0)	100.0 (0.0–100.0)	100.0 (100.0–100.0)
**AT**	1	100.0 (0.0–100.0)	100.0 (100.0–100.0)	100.0 (100.0–100.0)	100.0 (0.0–100.0)	100.0 (100.0–100.0)
**AV block**	3	100.0 (0.0–100.0)	99.8 (99.4–100.0)	99.8 (99.4–100.0)	75.0 (0.0–100.0)	100.0 (100.0–100.0)
**Others**	1	0.0 (0.0–0.0)	100.0 (100.0–100.0)	99.8 (99.4–100.0)	—	99.8 (99.4–100.0)

12L vs. 1L						
480 pairs. 194 participants						
	*n*	Sensitivity (95% CI)	Specificity (95% CI)	Accuracy (95% CI)	PPV (95% CI)	NPV (95% CI)
**Sinus rhythm**	413	99.3 (98.2–100.0)	88.1 (77.6–96.0)	97.7 (96.0–99.0)	98.1 (96.4–99.5)	95.2 (88.5–100.0)
**APC**	21	71.4 (41.2–96.4)	100.0 (100.0–100.0)	98.8 (97.1–99.8)	100.0 (100.0–100.0)	98.7 (97.0–99.8)
**VPC**	6	83.3 (0.0–100.0)	99.6 (98.7–100.0)	99.4 (98.4–100.0)	71.4 (0.0–100.0)	99.8 (99.3–100.0)
**AF**	35	97.1 (91.2–100.0)	100.0 (100.0–100.0)	99.8 (99.4–100.0)	100.0 (100.0–100.0)	99.8 (99.3–100.0)
**AFL**	1	0.0 (0.0–0.0)	100.0 (100.0–100.0)	99.8 (99.4–100.0)	—	99.8 (99.4–100.0)
**AT**	1	100.0 (0.0–100.0)	100.0 (100.0–100.0)	100.0 (100.0–100.0)	100.0 (0.0–100.0)	100.0 (100.0–100.0)
**AV block**	2	0.0 (0.0–0.0)	100.0 (100.0–100.0)	99.6 (98.7–100.0)	—	99.6 (98.7–100.0)
**Others**	1	0.0 (0.0–0.0)	100.0 (100.0–100.0)	99.8 (99.4–100.0)	—	99.8 (99.4–100.0)

Eighteen cases from 12-lead/single-lead pairs with a patient's rhythm appeared to have changed between the time the 12-lead ECG was obtained and the single-lead ECG was taken were excluded. Ungradable single-lead recordings were included as test failures (incorrect). 95% CIs were estimated using a non-parametric participant-level cluster bootstrap (1000 iterations), resampling participants with replacement to account for repeated measurements within participants.

ECG, electrocardiogram; 12L, 12-lead electrocardiogram; 6L, 6-lead handheld electrocardiogram; 1L, single-lead smartwatch electrocardiogram; CI, confidence interval; PPV, positive predictive value; NPV, negative predictive value; APC, atrial premature complex; VPC, ventricular premature complex; AF, atrial fibrillation; AFL, atrial flutter; AT, atrial tachycardia; AV block, atrioventricular block

The exploratory analysis showed a high diagnostic performance for device-automated interpretations for both devices. 6-lead ECG demonstrated an overall accuracy of 96.0% (93.9–97.5), with a sensitivity of 100.0% (90.3–100.0) and specificity of 99.8% (98.8–100.0) for AF or AFL classification. Single-lead ECG showed an overall accuracy of 90.8% (87.9–93.3), with sensitivity of 97.1% (84.7–99.9) and specificity of 98.0% (96.2–99.1) for AF classification.

### Numerical parameter assessment of consumer-grade ECG devices

In the ECG parameter comparisons, exclusions due to noise or labelling feasibility resulted in 485 12-lead/6-lead pairs (*n* = 193) and 467 12-lead/single-lead pairs (*n* = 190) (*Figure 3*). The parameter-specific sample sizes are summarized in *Table 3*.

**Table 3:** Agreement of numerical ECG parameters between standard 12-lead and consumer-grade device ECGs (Bland–Altman analysis and absolute mean differences)

12L vs. 6L						
Parameter	*n*	Mean difference (SD)	Upper LOA	Lower LOA	Outliers *n* (%)	Absolute mean difference (SD)
**Heart rate (bpm)**	479	−2.31 (0.86)	−0.62	−4.00	11 (2.3)	2.31 (0.86)
**PR interval (ms)**	448	4.90 (10.89)	26.24	−16.43	23 (5.13)	9.26 (7.52)
**QRS duration (ms)**	485	0.98 (10.90)	22.35	−20.39	21 (4.33)	8.63 (6.72)
**QT interval (ms)**	485	15.37 (17.32)	49.32	−18.57	25 (5.15)	19.14 (13.03)
**QTc interval (ms)**	479	10.10 (18.33)	46.02	−25.82	24 (5.01)	16.57 (12.77)
**P amplitude (mV)**	448	−0.01 (0.03)	0.04	−0.06	22 (4.91)	0.02 (0.02)
**QRS amplitude (mV)**	485	0.06 (0.08)	0.21	−0.10	15 (3.09)	0.07 (0.07)
**T amplitude (mV)**	485	0.01 (0.04)	0.10	−0.07	20 (4.12)	0.03 (0.03)

12L vs. 1L						
Parameter	*n*	Mean difference (SD)	Upper LOA	Lower LOA	Outliers *n* (%)	Absolute mean difference (SD)
**Heart rate (bpm)**	461	−0.29 (4.59)	8.70	−9.28	28 (6.07)	2.76 (3.67)
**PR interval (ms)**	430	21.04 (19.53)	59.32	−17.24	20 (4.65)	23.37 (16.68)
**QRS duration (ms)**	467	−4.25 (12.90)	21.05	−29.54	25 (5.35)	10.59 (8.46)
**QT interval (ms)**	467	1.94 (23.43)	47.87	−43.99	20 (4.28)	17.53 (15.78)
**QTc interval (ms)**	461	1.60 (26.43)	53.41	−50.20	23 (4.99)	19.95 (17.42)
**P amplitude (mV)**	430	0.02 (0.05)	0.11	−0.08	20 (4.65)	0.04 (0.04)
**QRS amplitude (mV)**	467	−0.03 (0.52)	0.98	−1.04	18 (3.85)	0.42 (0.30)
**T amplitude (mV)**	467	−0.05 (0.13)	0.20	−0.29	35 (7.49)	0.11 (0.08)

The bold box contains results from Bland–Altman analysis. LOA were computed as bias ± 1.96 × SD, where SD reflects the square root of the sum of the between-participant and within-participant variance components estimated from the mixed-effects model. Cases without P*-*wave were excluded from the PR interval and P amplitude analysis. Cases where non-consecutive beats were labelled were excluded from the heart rate and QTc interval analysis.

ECG, electrocardiogram; 12L, 12-lead electrocardiogram; 6L, 6-lead handheld electrocardiogram; 1L, single-lead smartwatch electrocardiogram; SD, standard deviation; LOA, limits of agreement; bpm, beats per minute; ms, milliseconds; mV, millivolts

**Figure 4:**
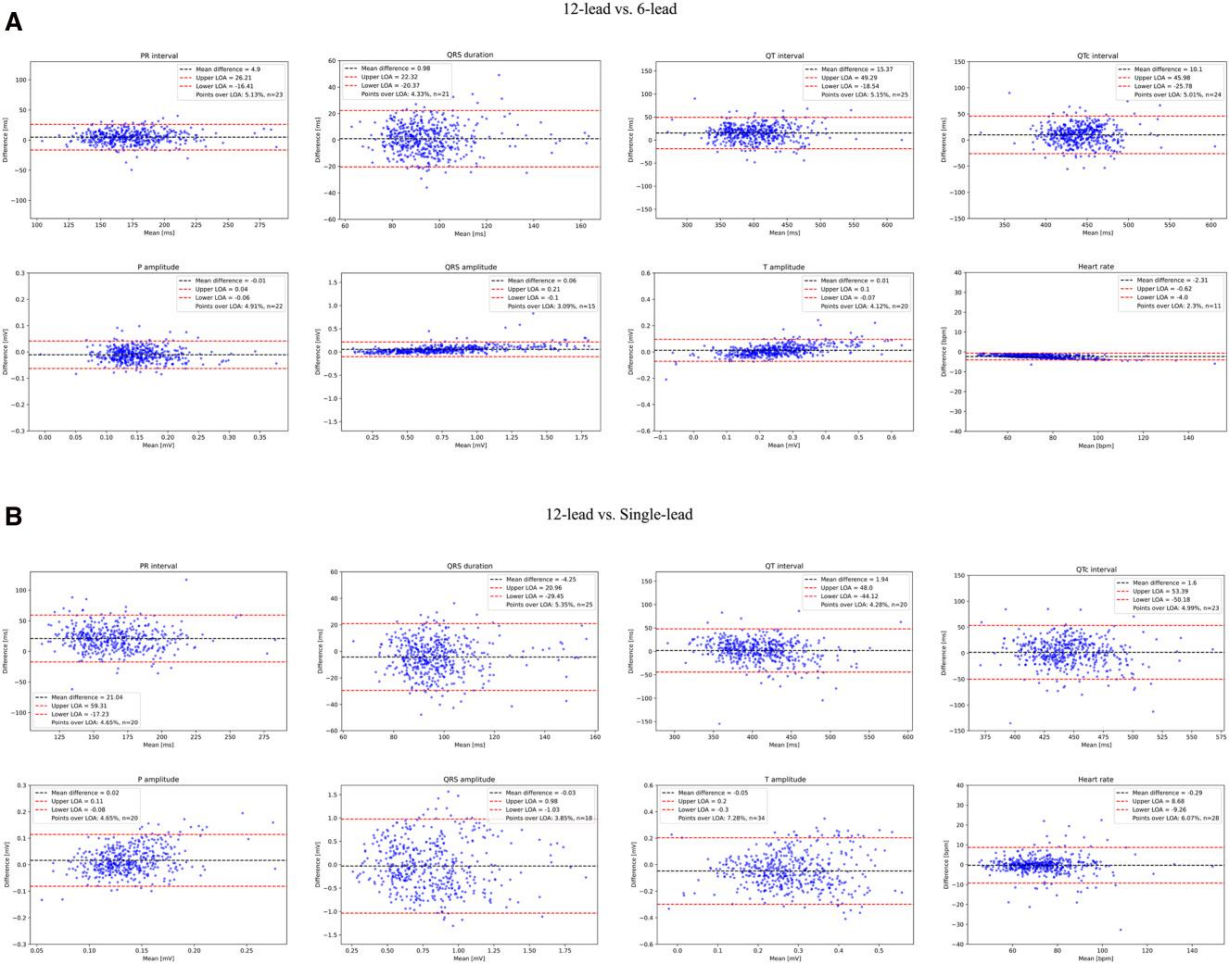
Bland–Altman plots comparing ECG parameters between 12-lead and consumer-grade device ECGs. Each panel shows Bland–Altman analysis of a specific ECG parameter comparing measurements from the 12-lead ECG and either the 6-lead ECG (top row) or single-lead ECG (bottom row). The y-axis represents the difference between the two measurements, while the x-axis represents the mean of the two. Red dashed lines indicate the 95% limits of agreement, computed as bias ± 1.96 × SD, where SD reflects the square root of the sum of the between-participant and within-participant variance components estimated from the mixed-effects model, and the black dashed line represents the mean difference. The parameters compared include PR interval, QRS duration, QT interval, QTc interval (Bazett's formula), P-wave amplitude, QRS amplitude, T-wave amplitude, and heart rate. The analysis demonstrates varying levels of agreement between 12-lead ECGs and consumer-grade devices, with amplitude measurements showing relatively wider dispersion, particularly for the single-lead ECG. ECG, electrocardiogram.

Agreement between the 12-lead ECG was assessed using the Bland–Altman analysis (*Table 3*; *Figure 4*). For interval measures, PR interval showed a smaller mean difference for the 6-lead ECG than the single-lead ECG (4.90 ms vs. 21.04 ms), while QT/QTc demonstrated different bias–variability profiles: the single-lead ECG had smaller mean bias (QT: 1.94 ms; QTc: 1.60 ms) but wider LOA (QT: −43.99 to 47.87 ms; QTc: −50.20 to 53.41 ms), whereas the 6-lead ECG had larger positive mean differences (QT: 15.37 ms; QTc: 10.10 ms) with narrower LOA (QT: −18.57 to 49.32 ms; QTc: −25.82 to 46.02 ms). For the amplitude measures, the largest contrast was observed for the QRS amplitude, with a substantially wider LOA for the single-lead ECG than for the 6-lead ECG.

Further analysis on the absolute mean differences is summarized in *Table 3*. For QT, the absolute mean difference was 19.14 ms for the 6-lead ECG and 17.53 ms for the single-lead ECG, whereas for QTc, it was 16.57 ms for the 6-lead ECG and 19.95 ms for the single-lead ECG. The other parameters showed similar patterns, particularly the amplitude measures. In a patient-level sensitivity analysis, the Bland–Altman findings and QT/QTc bias estimates differed in magnitude; however, the overall patterns of agreement and between-device differences remained similar (see Supplementary material online, *Table S4*).

#### Intraclass correlation coefficient

The inter-device reliability assessed by ICC is shown in *Table 4*. The ICCs for heart rate and QT interval were similar between the devices, whereas larger between-device differences were observed for PR, QRS duration, QTc, and amplitude measures, particularly for QRS amplitude (*Table 4*). Overall, the findings were concordant in patient-level sensitivity analysis (one recording per participant) (see Supplementary material online, *Table S5*).

**Table 4:** Inter-device absolute agreement of numerical parameters with intraclass correlation coefficient (ICC)

Parameter	12L vs. 6L	12L vs. 1L
**Heart rate**	0.98 (0.98–0.99)	0.94 (0.91–0.96)
**PR interval**	0.89 (0.85–0.92)	0.57 (0.48–0.63)
**QRS duration**	0.73 (0.63–0.80)	0.59 (0.44–0.70)
**QT interval**	0.86 (0.83–0.88)	0.85 (0.81–0.89)
**QTc interval**	0.77 (0.72–0.81)	0.63 (0.56–0.70)
**P amplitude**	0.81 (0.73–0.86)	0.15 (0.01–0.27)
**QRS amplitude**	0.96 (0.94–0.97)	0.04 (−0.13–0.21)
**T amplitude**	0.92 (0.90–0.93)	0.36 (0.26–0.44)

Cases without P-wave were excluded from the PR interval and P amplitude analysis. Cases where non-consecutive beats were labelled were excluded from the heart rate and QTc interval analysis. ICC is presented with 95% confidence interval estimated via cluster bootstrapping.

ECG, electrocardiogram; 12L, 12-lead electrocardiogram; 6L, 6-lead handheld electrocardiogram; 1L, single-lead smartwatch electrocardiogram

#### Subgroup analysis

Subgroup analyses were conducted to explore diagnostic performance, and numerical agreement varied across the clinical and demographic subgroups (see Supplementary Material  *S3*). Overall, rhythm interpretation accuracy did not differ significantly across subgroups for either device (see Supplementary material online, *Table S6*). For numerical parameters, some subgroup comparisons of amplitude measures reached statistical significance; however, the magnitudes of the differences were small, and these analyses were interpreted as exploratory (see Supplementary material online, *Table S7*).

## Discussion

In this study, both the 6-lead handheld ECG and the single-lead smartwatch ECG were evaluated against the standard 12-lead ECG, with the 6-lead device demonstrating closer agreement across rhythm classification and quantitative parameter assessment. For rhythm classification, the 6-lead device showed higher sensitivity for ectopic beats, AFL, and AV block, whereas both devices performed well for AF detection, in line with previous reports.^16,17^

The exploratory analysis similarly demonstrated the high diagnostic performance of automated device interpretations for both devices, with the 6-lead device outperforming the single-lead device. Beyond rhythm interpretation, the 6-lead device showed closer agreement with the 12-lead ECG across numerical parameters, including heart rate, PR interval, QRS duration, and QTc interval. This advantage was particularly evident in the measurement of waveform amplitudes. The 6-lead configuration reliably captured the P-wave, QRS, and T-wave amplitudes with close concordance to the standard ECG, enabling the robust detection of low-amplitude atrial signals that are critical for rhythm discrimination. By contrast, the single-lead device demonstrated greater variability and limited reproducibility across these measurements, thereby reducing its clinical reliability. Practically, these three ECG modalities play complementary roles. Single-lead ECG is suitable for screening and atrial fibrillation detection, 6-lead ECG adds value for more detailed rhythm and interval assessment, and standard 12-lead ECG remains mandatory for comprehensive evaluation, including ischaemia and complex conduction abnormalities. While our findings suggest a closer agreement between the 6-lead configuration and the 12-lead ECG, these results were derived from specific devices and may not be uniformly generalizable to all platforms. Device-specific differences in the signal processing and diagnostic performance have been reported in previous technical and clinical studies.^18,19^

### Main findings

Our study demonstrated a strong concordance between the 6-lead and 12-lead ECG systems in rhythm classification and interval measurement. These findings are consistent with prior studies demonstrating the incremental value of multilead configurations, as reported by Azram *et al*. and Kleiman *et al*.^9,20^ However, these studies did not employ simultaneous acquisition, which distinguishes the novelty of our study. Similarly, Scholten *et al*. demonstrated superior discrimination of AF and AFL using a 6-lead device,^8^ and Bacevicius *et al*. reported improved performance of 6-lead ECG in patients with APCs and VPCs^17^—findings that is consistent with our results.

Nevertheless, some studies have reported that single-lead ECG devices can provide reliable interval measurements beyond AF detection, particularly for QTc estimation. Marín *et al*. demonstrated excellent agreement between single-lead and 12-lead QTc measurements,^21^ and Beers *et al*. reported clinically acceptable QTc estimation in primary care,^22^ both studies were conducted mainly in patients with a normal QTc interval. While these results highlight scenarios in which single-lead recordings may be sufficient, in our study of patients with similar baseline QTc intervals, the 6-lead ECG provided a more consistent estimation than the single-lead ECG. The 6-lead ECG exhibited a mean QTc difference of 10.10 ms with LOA of −25.82 to 46.02 ms. In contrast, the single-lead ECG showed a negligible mean difference of 1.60 ms but wider LOA (−50.20 to 53.41 ms), along with a mean absolute difference of 19.95 ms and an ICC of 0.63, indicating substantial random variability. Although the single-lead ECG demonstrated a relatively small mean bias in the QT/QTc measurements, it exhibited a substantially wider LOA, reflecting greater random variability. In contrast, the 6-lead ECG showed a modest systematic offset but narrower limits of agreement, indicating more consistent measurements. From a clinical standpoint, systematic bias may be corrected through calibration; however, high random variability may limit reproducibility at the individual level. This suggests limitations of single-lead technology, which provides only a lead I like vector, whereas the 6-lead includes lead II and offers more reliable QTc estimation.^23^ However, Giudicessi *et al*. demonstrated that artificial intelligence (AI)-enabled QTc prediction using single-lead mobile ECG could overcome these limitations, indicating advanced algorithms may enhance single-lead performance.^10^

Furthermore, the P-wave amplitude was a key finding of our study. The 6-lead configuration showed minimal mean difference from the 12-lead (−0.01 mV) with narrow LOA (−0.06 to 0.04 mV) and strong reliability (ICC = 0.81, 95% CI: 0.73–0.86). In contrast, the single-lead demonstrated a slightly larger mean difference (0.02 mV) with wider LOA (−0.08 to 0.11 mV) and very poor agreement (ICC = 0.15, 95% CI: 0.01–0.27), indicating that individual measurements were largely random despite a modest average bias. Absolute differences further emphasized this discrepancy: the 6-lead exhibited a mean absolute difference of 0.02 mV (SD 0.02), whereas the single-lead showed a larger mean absolute difference of 0.04 mV (SD 0.04). These results highlight the distinct advantages of the 6-lead device for reliably capturing low-amplitude atrial signals, which are often critical for rhythm classification. However, these findings should not be extrapolated to broader diagnostic indications that require precordial lead information from standard 12-lead ECG. Given its performance, the 6-lead device may serve as an adjunct tool to support structured rhythm assessments and interval measurements in selected clinical settings.

Finally, subgroup analyses demonstrated that the diagnostic accuracy and numerical agreement of the 6-lead ECG with the standard 12-lead ECG were consistently maintained across all clinical subgroups. The diagnostic accuracy ranged from 96% to 100%, with no significant differences according to age, sex, left atrial size, or body composition. Minor variations in the amplitude parameters were statistically significant but clinically negligible (<0.1 mV), indicating stable waveform fidelity and reliable rhythm interpretation. In contrast, single-lead ECG showed greater subgroup-dependent variability, with lower diagnostic accuracy and larger amplitude differences across the subgroups.

### Potential mechanisms

Several mechanisms can explain these observations. The additional limb leads in a 6-lead configuration enhance the spatial resolution and electrical axis coverage, improving the likelihood of capturing the maximal deflection of both atrial and ventricular activity. This broader sampling increases the visibility of low-amplitude waveforms, particularly the P-wave, which is critical for rhythm discrimination. By contrast, a single-lead device provides only one electrical vector that may not optimally align with the predominant QRS axis of the patient, resulting in voltage attenuation. The underestimation of the QRS amplitude in single-lead recordings is likely compounded by differences in electrode placement, signal processing algorithms, and filtering parameters, which may attenuate high-frequency components or peak voltages. Additionally, the simultaneous acquisition of 6-lead and 12-lead ECGs minimizes physiological variability, whereas the sequential acquisition required for smartwatches introduces potential differences in heart rate, autonomic tone, or rhythm stability. Motion artefacts and baseline wandering are also more likely in smartwatch recordings, further contributing to amplitude and interval variability.

### Strengths of the study

This study has several strengths. The prospective design, simultaneous acquisition of 6-lead and 12-lead ECGs, and blinded, independent review by two electrophysiology specialists enhanced internal validity. The use of multiple complementary statistical methods provides a robust assessment of the agreement. Importantly, the relevance of our work extends beyond AF detection, the predominant focus of earlier validation studies,^1,2,24^ as it showed consistent performance not only for AF but also for other arrhythmias. By comprehensively assessing waveform amplitudes and interval reproducibility in paired simultaneous recordings, our study demonstrates that 6-lead ECGs provide more precise and reliable measurements across both atrial and ventricular signals, thereby contributing to more accurate rhythm classification. Additionally, the handheld design highlights its practical usability and facilitates convenient screening. This enables the early detection of arrhythmias in clinical and community settings. Finally, subgroup analyses further strengthened the study by confirming the robustness of diagnostic accuracy and waveform agreement across diverse patient characteristics.

## Limitations

The limitations include the single-centre design, which may limit generalizability, and the exclusion of noisy or ungradable ECGs from numerical analyses, which may reflect optimized acquisition conditions and potentially overestimate device performance compared with unsupervised real-world use.

For rhythm interpretation, ungradable single-lead recordings were classified as incorrect in the primary analysis (intention-to-diagnose approach). This handling may underestimate single-lead performance in settings where repeat acquisition is feasible. However, as these devices are primarily used as patient-driven event recorders, repeat acquisition may not always be possible, given that the symptomatic episode may have already resolved by the time recording quality is assessed.

Because the study population was derived from a tertiary arrhythmia clinic with a relatively high prevalence of AF, the observed predictive values and diagnostic performance may not be directly generalizable to lower-risk or screening populations. Differences in device sampling rates and filtering specifications may have contributed to the measurement discrepancies. Moreover, larger left atrial size is associated with lower P-wave amplitude due to more dispersed and attenuated atrial electrical activity,^25^ P-wave amplitude could not be measured in our patients with persistent AF, as P-waves were absent, which may have led to an underestimation of this effect. Although rhythm-discordant pairs attributed to the acquisition time gap were excluded from the primary analysis, the single-lead ECG was acquired sequentially rather than simultaneously, allowing rhythm changes to occur between recordings. This sequential design has an inherent limitation within the three-device comparison framework and may have influenced comparative diagnostic performance. However, numerical ECG parameters such as heart rate, PR interval, QRS duration, QT/QTc interval, and waveform amplitudes are less dependent on transient rhythm variation, and therefore remain appropriate for comparative analysis despite sequential acquisition. Additionally, the amplitude measurements for the 12-lead and 6-lead ECGs were derived from lead II, whereas the single-lead ECG corresponded to lead I. The use of different reference leads may have influenced the inter-device amplitude comparisons. However, the availability of multiple reference leads in the 6-lead ECG allows selection of the lead with the most clearly identifiable P-wave signal (such as lead II), which may facilitate more reliable amplitude measurements. Furthermore, for rhythm interpretation, all available leads for each device were used rather than a single predefined lead, allowing rhythm classification to align with the intended clinical use of each device. Although expert readers were blinded to the clinical information and cross-device interpretations, blinding to the ECG modality was not feasible because of the distinct visual formats of the recordings.

## Conclusions

The 6-lead ECG demonstrated closer agreement with the standard 12-lead ECG than with the single-lead device in terms of rhythm classification and waveform measurements. These findings suggest that handheld 6-lead ECGs may provide additional information for rhythm and interval assessments. Further studies incorporating uniform lead selection and full simultaneous acquisition across devices are required.

## Supplementary Material

ztag086_Supplementary_Data

## Data Availability

The data on which this article is based are available in the article itself and in the Supplementary material. Due to intellectual property constraints, sharing the code and model weights is restricted. However, restricted access to statistical analysis for verified researchers may be provided upon request for research purposes.
