# Supplementary material for: Comparative analysis of 6-lead and single-lead consumer-grade electrocardiograms: diagnostic accuracy, numerical agreement, and inter-rater reliability
Source: Eur Heart J Digit Health. 2026 Jun 11;7(6):ztag086. doi: 10.1093/ehjdh/ztag086 (PMC13403557; doi:10.1093/ehjdh/ztag086)

## Supplementary Material S1. Patient-level sensitivity analysis.

*Methods*

To evaluate robustness to repeated measurements, we conducted a patient-level sensitivity analysis using one pre-specified ECG pair per participant (first-visit pair), thereby ensuring independent observations. Participants were included only if they had valid and complete data for the corresponding outcome.

For rhythm interpretation, the 12-lead ECG served as the reference standard, and diagnostic performance metrics (accuracy, sensitivity, specificity, positive predictive value [PPV], and negative predictive value [NPV]) were calculated per rhythm category with 95% confidence intervals.

For numerical parameters, agreement was assessed using Bland–Altman analysis and absolute mean differences, and inter-device reliability was assessed using ICC with 95% confidence intervals. Participant counts varied by parameter according to the same pre-specified exclusions as in the main analysis (e.g., noise/labeling feasibility, absence of visible P waves for PR/P amplitude, and non-consecutive beat labeling for heart rate/QTc) and are reported in Supplementary Table S4.

*Results*

For the patient-level analysis, three participants were excluded due to missing ECG recordings. A total of 191 participants (191 pairs) were included for the 12-lead vs 6-lead comparison, and 186 participants (186 pairs) were included for the 12-lead vs single-lead comparison. In the single-lead comparison, five pairs with apparent rhythm discordance attributable to the acquisition time gap were excluded in accordance with the main analysis. The number of excluded discordant pairs differs from the main analysis because the patient-level analysis was restricted to one pre-specified recording per participant.

Overall, patient-level results were consistent with the main recording-level analyses. No 6-lead recordings were ungradable (0.0%), whereas three single-lead recordings were ungradable (1.61%), and these were treated as non-diagnostic test failures (counted as incorrect) in diagnostic performance calculations. Overall diagnostic accuracy was 98.4% (95% CI, 95.5–99.7) for the 6-lead ECG and 95.2% (91.0–97.8) for the single-lead ECG. In a paired comparison restricted to recordings with valid classifications for both devices (n=186), McNemar’s exact test (two-sided) did not show a statistically significant difference between devices (6-lead: 98.4% [95.4–99.7]; p=0.0703). Diagnostic performance metrics by rhythm category showed similar patterns to the main analysis (Supplementary Table S2).

Inter-rater agreement (Cohen’s kappa) was numerically lower in the patient-level analysis (12-lead: 0.85 [95% CI, 0.73–0.97]; 6-lead: 0.80 [0.67–0.93]; single-lead: 0.77 [0.64–0.90]), while the relative ordering across modalities remained the same (12-lead highest, followed by 6-lead and single-lead).

For numerical ECG parameters, 8 (4.19%), 3 (1.57%), and 10 (5.24%) pairs were excluded due to excessive noise or inability to reliably label beats on the 12-lead, 6-lead, and single-lead ECGs, respectively. Overall, findings were concordant with the main analyses: the 6-lead device showed narrower LOA and smaller absolute mean differences than the single-lead device for amplitude measures, with broadly similar relative patterns across interval measures (Supplementary Table S4). QT/QTc bias estimates differed in magnitude in the patient-level analysis; however, overall agreement patterns—considering LOA, absolute mean differences, and ICC—remained similar. Inter-device reliability assessed by ICC was also concordant with the main findings, including higher ICCs for amplitude measures for the 6-lead device compared with the single-lead device (Supplementary Table S5).

## Supplementary Material S2. Sensitivity analysis excluding ungradable recordings.

*Methods*

In the primary analysis, ungradable recordings were conservatively classified as incorrect to provide a real-world estimate of diagnostic performance (intention-to-diagnose). To assess the robustness of this assumption, we re-estimated diagnostic performance after excluding ungradable recordings. Sensitivity, specificity, overall accuracy, PPV and NPV were calculated for each rhythm category, with 95% confidence intervals estimated using the participant-level cluster bootstrapping method (1,000 iterations) described in the main Methods. A paired comparison of overall accuracy between the two devices was performed using McNemar's exact test on the intersection set of recordings with valid classifications for both devices.

*Results*

Five single-lead ECG recordings were ungradable (1.04%); no 6-lead recordings were ungradable. After exclusion, the analysis included 498 recordings (194 participants) for the 6-lead ECG and 475 recordings (192 participants) for the single-lead ECG.

The diagnostic performance estimates for both devices were mostly consistent with the primary analysis (Supplementary Table S3). Sensitivity remained higher for the 6-lead ECG than for the single-lead ECG for atrial premature complex, atrial flutter, and first-degree atrioventricular block, while ventricular premature complex became comparable between the two devices. Specificity remained high and comparable across rhythm categories. Overall diagnostic accuracy was 98.6% (95% CI, 97.4–99.6) for the 6-lead ECG and 97.9% (96.2–99.0) for the single-lead ECG. In the paired comparison on the common subset (n=475), McNemar's exact test did not show a statistically significant difference in overall accuracy (6-lead: 98.5% [97.0–99.4]; p=0.5811), consistent with the primary analysis. These findings indicate that our conclusions are robust to the handling of ungradable recordings.

## Supplementary Material S3. Subgroup analyses in rhythm interpretation and numeric parameter assessment.

To further investigate device performance under varying clinical conditions, subgroup analyses were performed. For these subgroup analyses, only the ECG pair (12-lead vs. 6-lead or 12-lead vs. single-lead) from each patient's first visit was utilized. This specific data selection strategy was adopted to avoid non-independence due-to repeated measures and ensure that the analysis primarily reflected the influence of individual patient characteristics on ECG parameters, rather than potential variations arising from repeated measurements across multiple visits or within a single visit. Furthermore, participants were included in a specific subgroup analysis only if they had valid and complete data for the respective subgroup variable. The following baseline characteristics were used to define subgroups: age (<65 vs. ≥65), sex (male vs. female), weight (<70 kg vs. ≥70 kg), Body Surface Area (BSA) categorized as low (<1.6 m^2^), normal (1.6–2.0 m^2^), and high (>2.0 m^2^), and Body Mass Index (BMI) classified as underweight to normal (<25.0 kg/m^2^) and overweight to obese (≥25.0 kg/m^2^). Additionally, echocardiography parameters including Left Ventricular Ejection Fraction (LVEF) (reduced to mildly reduced [<50%] vs. preserved EF [≥50%]), Left Atrial diameter (LA diameter) (normal [≤40 mm] vs. enlarged [>40 mm]), and Left Atrial Volume Index (LAVI) (normal [≤34 mL/m^2^] vs. enlarged [>34 mL/m^2^]) were used for subgroup stratification. Given the extensive number of subgroup analysis results for each ECG parameter, this subgroup analysis on numeric parameters focuses on presenting the findings for amplitude metrics, namely the P/QRS/T amplitudes.

For subgroup comparisons of diagnostic performance metrics (accuracy), we compared proportions between subgroups within each device (e.g., age <65 vs. ≥65) using the Chi-squared test or Fisher’s exact test, as appropriate. For comparisons of numerical ECG parameters, we compared the Bland-Altman mean differences between subgroups. For comparisons involving two subgroups, we used an independent t-test (or the Mann-Whitney U test when distributional assumptions were not met). For three subgroups, we used one-way analysis of variance (ANOVA) (or the Kruskal-Wallis test when the assumptions were not met). A p-value < 0.05 was considered statistically significant. Subgroup analyses were exploratory and p-values were not adjusted for multiple comparisons.

## Supplementary Table S1. Rhythm discordance attributable to acquisition time gap (excluded pairs)

| 12L rhythm | 1L rhythm | Pattern | Number of cases (%) |
| --- | --- | --- | --- |
| Sinus rhythm | APC | Transient atrial ectopy detected only on 1L | 13 (72.2%) |
| VPC | Sinus rhythm | Ventricular ectopy absent on later 1L recording | 2 (11.1%) |
| AF | Sinus rhythm | AF converted before 1L acquisition | 1 (5.6%) |
| APC | AF | New AF detected on later 1L recording | 1 (5.6%) |
| VPC | APC + VPC | Atrial ectopy additionally detected on 1L | 1 (5.6%) |

12-lead/single-lead pairs were excluded from the diagnostic performance assessment if a patient's rhythm appeared to have changed between the time the 12-lead ECG was obtained and when the single-lead ECG was performed. 18 cases from 12-lead/single-lead pairs were excluded.

12L, 12-lead; 6L, 6-lead; 1L, single-lead; APC, atrial premature complex; VPC, ventricular premature complex; AF, atrial fibrillation

## Supplementary Table S2. Sensitivity analysis on diagnostic performance of consumer-grade ECG devices compared to standard 12-lead ECG (patient-level analysis).

| **12L vs. 6L**  **191 pairs. 191 participants** | | | | | | |
| --- | --- | --- | --- | --- | --- | --- |
|  | **N** | **Sensitivity (95% CI)** | **Specificity (95% CI)** | **Accuracy (95% CI)** | **PPV (95% CI)** | **NPV (95% CI)** |
| **Sinus rhythm** | 172 | 98.8 (95.9−99.9) | 94.7 (74.0−99.9) | 98.4 (95.5−99.7) | 99.4 (96.8−100.0) | 90.0 (68.3−98.8) |
| **APC** | 7 | 100.0 (59.0−100.0) | 100.0 (98.0−100.0) | 100.0 (98.1−100.0) | 100.0 (59.0−100.0) | 100.0 (98.0−100.0) |
| **VPC** | 3 | 100.0 (29.2−100.0) | 100.0 (98.1−100.0) | 100.0 (98.1−100.0) | 100.0 (29.2−100.0) | 100.0 (98.1−100.0) |
| **AF** | 7 | 100.0 (59.0−100.0) | 99.5 (97.0−100.0) | 99.5 (97.1−100.0) | 87.5 (47.3−99.7) | 100.0 (98.0−100.0) |
| **AFL** | 0 | - | 100.0 (98.1−100.0) | 100.0 (98.1−100.0) | - | 100.0 (98.1−100.0) |
| **AT** | 0 | - | 100.0 (98.1−100.0) | 100.0 (98.1−100.0) | - | 100.0 (98.1−100.0) |
| **AV block** | 1 | 100.0 (2.5−100.0) | 99.5 (97.1−100.0) | 99.5 (97.1−100.0) | 50.0 (1.3−98.7) | 100.0 (98.1−100.0) |
| **Others** | 1 | 0.0 (0.0−97.5) | 100.0 (98.1−100.0) | 99.5 (97.1−100.0) | - | 99.5 (97.1−100.0) |
| **12L vs. 1L**  **186 pairs. 186 participants** | | | | | | |
|  | **N** | **Sensitivity (95% CI)** | **Specificity (95% CI)** | **Accuracy (95% CI)** | **PPV (95% CI)** | **NPV (95% CI)** |
| **Sinus rhythm** | 169 | 98.2 (94.9−99.6) | 70.6 (44.0−89.7) | 98.4 (95.5−99.7) | 97.1 (93.3−99.0) | 80.0 (51.9−95.7) |
| **APC** | 6 | 50.0 (11.8−88.2) | 100.0 (98.0−100.0) | 100.0 (98.1−100.0) | 100.0 (29.2−100.0) | 98.4 (95.3−99.7) |
| **VPC** | 2 | 50.0 (1.3−98.7) | 99.5 (97.0−100.0) | 100.0 (98.1−100.0) | 50.0 (1.3−98.7) | 99.5 (97.0−100.0) |
| **AF** | 7 | 100.0 (59.0−100.0) | 100.0 (98.0−100.0) | 99.5 (97.1−100.0) | 100.0 (59.0−100.0) | 100.0 (98.0−100.0) |
| **AFL** | 0 | - | 100.0 (98.0−100.0) | 100.0 (98.1−100.0) | - | 100.0 (98.0−100.0) |
| **AT** | 0 | - | 100.0 (98.0−100.0) | 100.0 (98.1−100.0) | - | 100.0 (98.0−100.0) |
| **AV block** | 1 | 0.0 (0.0−97.5) | 100.0 (98.0−100.0) | 99.5 (97.1−100.0) | - | 99.5 (97.0−100.0) |
| **Others** | 1 | 0.0 (0.0−97.5) | 100.0 (98.0−100.0) | 99.5 (97.1−100.0) | - | 99.5 (97.0−100.0) |

5 cases from 12-lead/single-lead pairs with a patient's rhythm appeared to have changed between the time the 12-lead ECG was obtained and the single-lead ECG was taken were excluded. 95% CIs were estimated using a Clopper-Pearson method.

ECG, electrocardiogram; 12L, 12-lead electrocardiogram; 6L, 6-lead handheld electrocardiogram; 1L, single-lead smartwatch electrocardiogram; CI, confidence interval; PPV, positive predictive value; NPV, negative predictive value; APC, sinus rhythm with atrial premature contraction; VPC, sinus rhythm with ventricular premature contraction; AF, atrial fibrillation; AFL, atrial flutter; AT, atrial tachycardia; AV block, atrioventricular block

## Supplementary Table S3. Sensitivity analysis on diagnostic performance of consumer-grade ECG devices compared to standard 12-lead ECG (excluding ungradable recordings).

| **12L vs. 6L**  **498 pairs. 194 participants** | | | | | | |
| --- | --- | --- | --- | --- | --- | --- |
|  | **N** | **Sensitivity (95% CI)** | **Specificity (95% CI)** | **Accuracy (95% CI)** | **PPV (95% CI)** | **NPV (95% CI)** |
| **Sinus rhythm** | 425 | 99.5 (98.8–100.0) | 93.2 (85.5–98.5) | 98.6 (97.4–99.6) | 98.8 (97.7–99.8) | 97.1 (93.1–100.0) |
| **APC** | 23 | 87.0 (68.2–100.0) | 100.0 (100.0–100.0) | 99.4 (98.6–100.0) | 100.0 (100.0–100.0) | 99.4 (98.5–100.0) |
| **VPC** | 9 | 100.0 (100.0–100.0) | 100.0 (100.0–100.0) | 100.0 (100.0–100.0) | 100.0 (100.0–100.0) | 100.0 (100.0–100.0) |
| **AF** | 35 | 97.1 (89.7–100.0) | 99.8 (99.3–100.0) | 99.6 (99.0–100.0) | 97.1 (91.7–100.0) | 99.8 (99.2–100.0) |
| **AFL** | 1 | 100.0 (0.0–100.0) | 100.0 (100.0–100.0) | 100.0 (100.0–100.0) | 100.0 (0.0–100.0) | 100.0 (100.0–100.0) |
| **AT** | 1 | 100.0 (0.0–100.0) | 100.0 (100.0–100.0) | 100.0 (100.0–100.0) | 100.0 (0.0–100.0) | 100.0 (100.0–100.0) |
| **AV block** | 3 | 100.0 (0.0–100.0) | 99.8 (99.4–100.0) | 99.8 (99.4–100.0) | 75.0 (0.0–100.0) | 100.0 (100.0–100.0) |
| **Others** | 1 | 0.0 (0.0–0.0) | 100.0 (100.0–100.0) | 99.8 (99.4–100.0) | - | 99.8 (99.4–100.0) |
| **12L vs. 1L**  **475 pairs. 192 participants** | | | | | | |
|  | **N** | **Sensitivity (95% CI)** | **Specificity (95% CI)** | **Accuracy (95% CI)** | **PPV (95% CI)** | **NPV (95% CI)** |
| **Sinus rhythm** | 411 | 99.8 (98.7–100.0) | 87.5 (76.8–94.4) | 98.1 (96.4–99.1) | 98.1 (96.3–99.2) | 98.2 (90.6–100.0) |
| **APC** | 19 | 78.9 (54.4–93.9) | 100.0 (99.2–100) | 99.2 (97.9–99.8) | 100.0 (78.2–100) | 99.1 (97.8–99.8) |
| **VPC** | 5 | 100.0 (47.8–100) | 99.6 (98.5–99.9) | 99.6 (98.5–99.9) | 71.4 (29.0–96.3) | 100.0 (99.2–100) |
| **AF** | 35 | 97.1 (85.1–99.9) | 100.0 (99.2–100) | 99.8 (98.8–100.0) | 100.0 (89.7–100) | 99.8 (98.7–100.0) |
| **AFL** | 1 | 0.0 (0–97.5) | 100.0 (99.2–100) | 99.8 (98.8–100.0) | - | 99.8 (98.8–100.0) |
| **AT** | 1 | 100.0 (2.5–100) | 100.0 (99.2–100) | 100.0 (99.2–100) | 100.0 (2.5–100) | 100.0 (99.2–100) |
| **AV block** | 2 | 0.0 (0–84.2) | 100.0 (99.2–100) | 99.6 (98.5–99.9) | - | 99.6 (98.5–99.9) |
| **Others** | 1 | 0.0 (0–97.5) | 100.0 (99.2–100) | 99.8 (98.8–100.0) | - | 99.8 (98.8–100.0) |

18 cases from 12-lead/single-lead pairs with a patient's rhythm appeared to have changed between the time the 12-lead ECG was obtained and the single-lead ECG was taken were excluded. Five ungradable single-lead recordings were excluded. 95% CIs were estimated using a non-parametric participant-level cluster bootstrap (1,000 iterations), resampling participants with replacement to account for repeated measurements within participants.

ECG, electrocardiogram; 12L, 12-lead electrocardiogram; 6L, 6-lead handheld electrocardiogram; 1L, single-lead smartwatch electrocardiogram; CI, confidence interval; PPV, positive predictive value; NPV, negative predictive value; APC, atrial premature complex; VPC, ventricular premature complex; AF, atrial fibrillation; AFL, atrial flutter; AT, atrial tachycardia; AV block, atrioventricular block

## Supplementary Table S4. Sensitivity analysis on agreement of numerical ECG parameters between standard 12-lead and consumer-grade device ECGs (Bland-Altman analysis and absolute mean differences; patient-level analysis).

| **12L vs. 6L** | | | | | | |
| --- | --- | --- | --- | --- | --- | --- |
| **Parameter** | **N** | **Mean difference (SD)** | **Upper LOA** | **Lower LOA** | **Outliers N (%)** | **Absolute mean difference (SD)** |
| **Heart rate (bpm)** | 178 | −1.45 (0.43) | −0.61 | −2.29 | 10 (5.62) | 1.45 (0.43) |
| **PR interval (ms)** | 173 | 3.30 (11.77) | 26.36 | −19.77 | 8 (4.62) | 9.16 (8.11) |
| **QRS duration (ms)** | 180 | −4.63 (9.88) | 14.74 | −24.00 | 10 (5.56) | 8.74 (6.55) |
| **QT interval (ms)** | 180 | 5.45 (15.93) | 36.68 | −25.77 | 6 (3.33) | 13.71 (9.79) |
| **QTc interval (ms)** | 178 | 1.55 (17.26) | 35.38 | −32.28 | 6 (3.37) | 13.87 (10.41) |
| **P amplitude (mV)** | 173 | 0.00 (0.03) | 0.05 | −0.05 | 7 (4.05) | 0.02 (0.02) |
| **QRS amplitude (mV)** | 180 | 0.05 (0.10) | 0.26 | −0.15 | 7 (3.89) | 0.06 (0.10) |
| **T amplitude (mV)** | 180 | 0.02 (0.05) | 0.12 | −0.08 | 6 (3.33) | 0.04 (0.04) |
| **12L vs. 1L** | | | | | | |
| **Parameter** | **N** | **Mean difference (SD)** | **Upper LOA** | **Lower LOA** | **Outliers N (%)** | **Absolute mean difference (SD)** |
| **Heart rate (bpm)** | 171 | −0.55 (3.81) | 6.91 | −8.01 | 10 (5.85) | 2.50 (2.93) |
| **PR interval (ms)** | 166 | 23.06 (19.75) | 61.78 | −15.66 | 8 (4.82) | 25.56 (16.44) |
| **QRS duration (ms)** | 173 | −4.91 (12.99) | 20.56 | −30.38 | 12 (6.94) | 10.96 (8.56) |
| **QT interval (ms)** | 173 | 3.98 (18.21) | 39.66 | −31.71 | 7 (4.05) | 14.35 (11.92) |
| **QTc interval (ms)** | 171 | 2.90 (21.35) | 44.74 | −38.95 | 9 (5.26) | 16.80 (13.53) |
| **P amplitude (mV)** | 166 | 0.02 (0.05) | 0.12 | −0.09 | 8 (4.82) | 0.04 (0.03) |
| **QRS amplitude (mV)** | 173 | −0.02 (0.54) | 1.04 | −1.07 | 6 (3.47) | 0.44 (0.30) |
| **T amplitude (mV)** | 173 | −0.05 (0.12) | 0.19 | −0.29 | 12 (6.94) | 0.11 (0.08) |

The bold box contains results from Bland-Altman analysis. LOA were computed as bias ± 1.96 × SD. Cases without P wave were excluded from the PR interval and P amplitude analysis. Cases where non-consecutive beats were labeled were excluded from the heart rate and QTc interval analysis.

ECG, electrocardiogram; 12L, 12-lead electrocardiogram; 6L, 6-lead handheld electrocardiogram; 1L, single-lead smartwatch electrocardiogram; SD, standard deviation; LOA, limits of agreement; bpm, beats per minute; ms, milliseconds; mV, millivolts

## Supplementary Table S5. Sensitivity analysis on inter-device absolute agreement of numerical parameters with intraclass correlation coefficient (patient-level analysis).

| **Parameter** | **12L vs. 6L** | **12L vs. 1L** |
| --- | --- | --- |
| **Heart rate** | 0.99 (0.24−1.00) | 0.95 (0.93−0.96) |
| **PR interval** | 0.87 (0.82−0.91) | 0.50 (−0.05−0.76) |
| **QRS duration** | 0.75 (0.60−0.83) | 0.54 (0.39−0.65) |
| **QT interval** | 0.91 (0.87−0.94) | 0.90 (0.87−0.93) |
| **QTc interval** | 0.81 (0.75−0.85) | 0.72 (0.64−0.79) |
| **P amplitude** | 0.82 (0.77−0.87) | −0.00 (−0.14−0.14) |
| **QRS amplitude** | 0.95 (0.90−0.97) | 0.01 (−0.14−0.16) |
| **T amplitude** | 0.90 (0.85−0.93) | 0.43 (0.27−0.56) |

Cases without P wave were excluded from the PR interval and P amplitude analysis. Cases where non-consecutive beats were labeled were excluded from the heart rate and QTc interval analysis. ICC is presented with 95% confidence interval.

ECG, electrocardiogram; 12L, 12-lead electrocardiogram; 6L, 6-lead handheld electrocardiogram; 1L, single-lead smartwatch electrocardiogram

## Supplementary Table S6. Subgroup analyses in rhythm interpretation comparing overall accuracy between consumer-grade ECG devices to standard 12-lead ECG.

| **Age** | **6L  Accuracy (95% CI)** | **12L N** | **6L N** | **P-value** | **1L  Accuracy (95% CI)** | **12L N** | **1L N** | **P-value** |
| --- | --- | --- | --- | --- | --- | --- | --- | --- |
| **<65** | 98.4 (94.5–99.8) | 128 | 126 |  | 96.8 (92.1–99.1) | 126 | 122 |  |
| **≥65** | 98.4 (91.5–100.0) | 63 | 62 | >.05 | 91.7 (81.6–97.2) | 60 | 55 | >.05 |
| **Sex** | **6L  Accuracy (95% CI)** | **12L N** | **6L N** | **P-value** | **1L  Accuracy (95% CI)** | **12L N** | **1L N** | **P-value** |
| **Male** | 98.5 (94.8–99.8) | 135 | 133 |  | 96.3 (91.6–98.8) | 135 | 130 |  |
| **Female** | 98.2 (90.4–100.0) | 56 | 55 | >.05 | 92.2 (81.1–97.8) | 51 | 47 | >.05 |
| **Weight (kg)** | **6L  Accuracy (95% CI)** | **12L N** | **6L N** | **P-value** | **1L  Accuracy (95% CI)** | **12L N** | **1L N** | **P-value** |
| **<70** | 97.8 (92.2–99.7) | 90 | 88 |  | 91.9 (83.9–96.7) | 86 | 79 |  |
| **≥70** | 99.0 (94.6–100.0) | 100 | 99 | >.05 | 98.0 (92.9–99.8) | 99 | 97 | >.05 |
| **BSA (m^2^)** | **6L  Accuracy (95% CI)** | **12L N** | **6L N** | **P-value** | **1L  Accuracy (95% CI)** | **12L N** | **1L N** | **P-value** |
| **Low BSA (<1.6)** | 96.4 (81.7–99.9) | 28 | 27 |  | 88.5 (69.8–97.6) | 26 | 23 |  |
| **Normal BSA (1.6**–**2.0)** | 99.2 (95.8–100.0) | 130 | 129 |  | 96.1 (91.1–98.7) | 127 | 122 |  |
| **High BSA (>2.0)** | 96.9 (83.8–99.9) | 32 | 31 | >.05 | 96.9 (83.8–99.9) | 32 | 31 | >.05 |
| **BMI (kg/m^2^)** | **6L  Accuracy (95% CI)** | **12L N** | **6L N** | **P-value** | **1L  Accuracy (95% CI)** | **12L N** | **1L N** | **P-value** |
| **Underweight to Normal (<25.0)** | 97.8 (92.3–99.7) | 91 | 89 |  | 93.2 (85.7–97.5) | 88 | 82 |  |
| **Overweight to obese (≥25.0)** | 99.0 (94.5–100.0) | 99 | 98 | >.05 | 96.9 (91.2–99.4) | 97 | 94 | >.05 |
| **LVEF (%)** | **6L  Accuracy (95% CI)** | **12L N** | **6L N** | **P-value** | **1L  Accuracy (95% CI)** | **12L N** | **1L N** | **P-value** |
| **Reduced to mildly reduced EF (<50)** | 100.0 (29.2–100.0) | 3 | 3 |  | 100.0 (29.2–100.0) | 3 | 3 |  |
| **Preserved EF (≥50)** | 98.8 (95.8–99.9) | 169 | 167 | >.05 | 95.7 (91.4–98.3) | 164 | 157 | >.05 |
| **LA diameter (mm)** | **6L  Accuracy (95% CI)** | **12L N** | **6L N** | **P-value** | **1L  Accuracy (95% CI)** | **12L N** | **1L N** | **P-value** |
| **Normal (≤40)** | 98.9 (93.9–100.0) | 89 | 88 |  | 94.1 (86.8–98.1) | 85 | 80 |  |
| **Enlarged (>40)** | 98.8 (93.3–100.0) | 81 | 80 | >.05 | 97.5 (91.3–99.7) | 80 | 78 | >.05 |
| **LAVI (ml/m^2^)** | **6L  Accuracy (95% CI)** | **12L N** | **6L N** | **P-value** | **1L  Accuracy (95% CI)** | **12L N** | **1L N** | **P-value** |
| **Normal (≤34)** | 100.0 (96.0–100.0) | 91 | 91 |  | 98.9 (94.0–100.0) | 90 | 89 |  |
| **Enlarged (>34)** | 98.6 (92.6–100.0) | 73 | 72 | >.05 | 94.3 (86.0–98.4) | 70 | 66 | >.05 |

For subgroup analyses, only the last acquired ECG pair (12-lead vs. 6-lead or 12-lead vs. single-lead) from each patient's first visit was utilized. This specific data selection strategy was adopted to ensure that the analysis primarily reflected the influence of individual patient characteristics on ECG parameters, rather than potential variations arising from repeated measurements across multiple visits or within a single visit. Furthermore, participants were included in a specific subgroup analysis only if they had valid and complete data for the respective subgroup variable. Fisher’s exact test or Chi-squared tests were employed to test statistical difference in overall accuracy between subgroups. A p-value < 0.05 was considered statistically significant. Subgroup analyses were exploratory and p-values were not adjusted for multiple comparisons.

ECG, electrocardiogram; 12L, 12-lead electrocardiogram; 6L, 6-lead handheld electrocardiogram; 1L, single-lead smartwatch electrocardiogram; BSA, body surface area; BMI, body mass index; LVEF, left ventricular ejection fraction; LA diameter, left atrial diameter; LAVI, left atrial volume index

## Supplementary Table S7. Subgroup Bland-Altman analyses in comparing numerical parameters of consumer-grade ECG devices to standard 12-lead ECG.

| **12L vs. 6L** | | | | | | | | | |
| --- | --- | --- | --- | --- | --- | --- | --- | --- | --- |
| **Parameter** | **Variable** | **Subgroup** | **N** | **Mean difference** | **SD** | **Upper LOA** | **Lower LOA** | **Outliers N (%)** | **P-value** |
| **P amplitude** | **Age** | **<65** | 117 | 0.01 | 0.03 | 0.07 | −0.05 | 5 (4.27%) |  |
|  |  | **≥65** | 56 | −0.01 | 0.03 | 0.05 | −0.07 | 1 (1.79%) | <.05 |
|  | **Sex** | **Male** | 123 | 0.00 | 0.03 | 0.06 | −0.06 | 3 (2.44%) |  |
|  |  | **Female** | 50 | 0.00 | 0.03 | 0.06 | −0.06 | 1 (2.0%) | >.05 |
|  | **Weight (kg)** | **<70** | 83 | 0.00 | 0.03 | 0.06 | −0.06 | 1 (1.2%) |  |
|  |  | **≥70** | 89 | 0.00 | 0.03 | 0.06 | −0.06 | 3 (3.37%) | >.05 |
|  | **BSA (m^2^)** | **Low BSA (<1.6)** | 23 | 0.00 | 0.02 | 0.04 | −0.04 | 4 (17.39%) |  |
|  |  | **Normal BSA (1.6**–**2.0)** | 122 | 0.00 | 0.03 | 0.06 | −0.06 | 3 (2.46%) |  |
|  |  | **High BSA (>2.0)** | 27 | 0.00 | 0.02 | 0.04 | −0.04 | 2 (7.41%) | >.05 |
|  | **BMI (kg/m^2^)** | **Underweight to normal (<25.0)** | 85 | 0.00 | 0.03 | 0.06 | −0.06 | 1 (1.18%) |  |
|  |  | **Overweight to obese (≥25.0)** | 87 | 0.00 | 0.03 | 0.06 | −0.06 | 3 (3.45%) | >.05 |
|  | **LVEF (%)** | **Reduced to mildly reduced EF (<50)** | 3 | 0.00 | 0.01 | 0.02 | −0.02 | 0 (0.0%) |  |
|  |  | **Preserved EF (≥50)** | 156 | 0.00 | 0.03 | 0.06 | −0.06 | 4 (2.56%) | >.05 |
|  | **LA diameter (mm)** | **Normal (≤40)** | 86 | 0.01 | 0.03 | 0.07 | −0.05 | 4 (4.65%) |  |
|  |  | **Enlarged (>40)** | 71 | 0.00 | 0.02 | 0.04 | −0.04 | 4 (5.63%) | >.05 |
|  | **LAVI (ml/m^2^)** | **Normal (≤34)** | 86 | 0.00 | 0.03 | 0.06 | −0.06 | 2 (2.33%) |  |
|  |  | **Enlarged (>34)** | 65 | 0.00 | 0.03 | 0.06 | −0.06 | 2 (3.08%) | >.05 |
| **QRS amplitude** | **Age** | **<65** | 121 | 0.05 | 0.10 | 0.25 | −0.15 | 4 (3.31%) |  |
|  |  | **≥65** | 59 | 0.05 | 0.10 | 0.25 | −0.15 | 3 (5.08%) | >.05 |
|  | **Sex** | **Male** | 128 | 0.05 | 0.11 | 0.27 | −0.17 | 4 (3.12%) |  |
|  |  | **Female** | 52 | 0.05 | 0.08 | 0.21 | −0.11 | 2 (3.85%) | >.05 |
|  | **Weight (kg)** | **<70** | 85 | 0.06 | 0.10 | 0.26 | −0.14 | 4 (4.71%) |  |
|  |  | **≥70** | 94 | 0.04 | 0.11 | 0.26 | −0.18 | 2 (2.13%) | >.05 |
|  | **BSA (m^2^)** | **Low BSA (<1.6)** | 25 | 0.07 | 0.10 | 0.27 | −0.13 | 1 (4.0%) |  |
|  |  | **Normal BSA (1.6**–**2.0)** | 126 | 0.05 | 0.11 | 0.27 | −0.17 | 5 (3.97%) |  |
|  |  | **High BSA (>2.0)** | 28 | 0.02 | 0.04 | 0.10 | −0.06 | 0 (0.0%) | >.05 |
|  | **BMI (kg/m^2^)** | **Underweight to normal (<25.0)** | 87 | 0.07 | 0.10 | 0.27 | −0.13 | 4 (4.60%) |  |
|  |  | **Overweight to obese (≥25.0)** | 92 | 0.03 | 0.10 | 0.23 | −0.17 | 3 (3.26%) | <.05 |
|  | **LVEF (%)** | **Reduced to mildly reduced EF (<50)** | 3 | 0.02 | 0.04 | 0.10 | −0.06 | 0 (0.0%) |  |
|  |  | **Preserved EF (≥50)** | 161 | 0.05 | 0.11 | 0.27 | −0.17 | 6 (3.73%) | >.05 |
|  | **LA diameter (mm)** | **Normal (≤40)** | 87 | 0.06 | 0.1 | 0.26 | −0.14 | 3 (3.45%) |  |
|  |  | **Enlarged (>40)** | 75 | 0.03 | 0.07 | 0.17 | −0.11 | 3 (4.0%) | <.05 |
|  | **LAVI (ml/m^2^)** | **Normal (≤34)** | 86 | 0.04 | 0.08 | 0.20 | −0.12 | 2 (2.33%) |  |
|  |  | **Enlarged (>34)** | 70 | 0.05 | 0.14 | 0.32 | −0.22 | 3 (4.29%) | >.05 |
| **T amplitude** | **Age** | **<65** | 121 | 0.02 | 0.05 | 0.12 | −0.08 | 3 (2.48%) |  |
|  |  | **≥65** | 59 | 0.02 | 0.05 | 0.12 | −0.08 | 2 (3.39%) | >.05 |
|  | **Sex** | **Male** | 128 | 0.02 | 0.05 | 0.12 | −0.08 | 4 (3.12%) |  |
|  |  | **Female** | 52 | 0.01 | 0.04 | 0.09 | −0.07 | 4 (7.69%) | <.05 |
|  | **Weight (kg)** | **<70** | 85 | 0.02 | 0.04 | 0.10 | −0.06 | 5 (5.88%) |  |
|  |  | **≥70** | 94 | 0.02 | 0.06 | 0.14 | −0.10 | 4 (4.26%) | >.05 |
|  | **BSA (m^2^)** | **Low BSA (<1.6)** | 25 | 0.00 | 0.05 | 0.10 | −0.10 | 1 (4.0%) |  |
|  |  | **Normal BSA (1.6**–**2.0)** | 126 | 0.02 | 0.05 | 0.12 | −0.08 | 4 (3.17%) |  |
|  |  | **High BSA (>2.0)** | 28 | 0.02 | 0.03 | 0.08 | −0.04 | 2 (7.14%) | >.05 |
|  | **BMI (kg/m^2^)** | **Underweight to normal (<25.0)** | 87 | 0.02 | 0.05 | 0.12 | −0.08 | 2 (2.3%) |  |
|  |  | **Overweight to obese (≥25.0)** | 92 | 0.02 | 0.05 | 0.12 | −0.08 | 3 (3.26%) | >.05 |
|  | **LVEF (%)** | **Reduced to mildly reduced EF (<50)** | 3 | 0.02 | 0.07 | 0.16 | −0.12 | 0 (0.0%) |  |
|  |  | **Preserved EF (≥50)** | 161 | 0.02 | 0.05 | 0.12 | −0.08 | 5 (3.11%) | >.05 |
|  | **LA diameter (mm)** | **Normal (≤40)** | 87 | 0.02 | 0.05 | 0.12 | −0.08 | 2 (2.3%) |  |
|  |  | **Enlarged (>40)** | 75 | 0.01 | 0.05 | 0.11 | −0.09 | 3 (4.0%) | >.05 |
|  | **LAVI (ml/m2)** | **Normal (≤34)** | 86 | 0.02 | 0.04 | 0.10 | −0.06 | 2 (2.33%) |  |
|  |  | **Enlarged (>34)** | 70 | 0.02 | 0.06 | 0.14 | −0.10 | 4 (5.71%) | >.05 |
| **12L vs. 1L** | | | | | | | | | |
| **Parameter** | **Variable** | **Subgroup** | **N** | **Mean difference** | **SD** | **Upper LOA** | **Lower LOA** | **Outliers N (%)** | **P-value** |
| **P amplitude** | **Age** | **<65** | 112 | 0.02 | 0.05 | 0.12 | −0.08 | 6 (5.36%) |  |
|  |  | **≥65** | 54 | 0.00 | 0.05 | 0.10 | −0.10 | 1 (1.85%) | <.05 |
|  | **Sex** | **Male** | 117 | 0.02 | 0.05 | 0.12 | −0.08 | 5 (4.27%) |  |
|  |  | **Female** | 49 | 0.02 | 0.05 | 0.12 | −0.08 | 5 (10.20%) | >.05 |
|  | **Weight (kg)** | **<70** | 81 | 0.03 | 0.05 | 0.13 | −0.07 | 5 (6.17%) |  |
|  |  | **≥70** | 84 | 0.01 | 0.05 | 0.11 | −0.09 | 5 (5.95%) | <.05 |
|  | **BSA (m^2^)** | **Low BSA (<1.6)** | 23 | 0.02 | 0.05 | 0.12 | −0.08 | 3 (13.04%) |  |
|  |  | **Normal BSA (1.6**–**2.0)** | 115 | 0.02 | 0.05 | 0.12 | −0.08 | 6 (5.22%) |  |
|  |  | **High BSA (>2.0)** | 27 | 0.00 | 0.04 | 0.08 | −0.08 | 2 (7.41%) | >.05 |
|  | **BMI (kg/m^2^)** | **Underweight to normal (<25.0)** | 79 | 0.03 | 0.05 | 0.13 | −0.07 | 5 (6.33%) |  |
|  |  | **Overweight to obese (≥25.0)** | 86 | 0.00 | 0.05 | 0.10 | −0.10 | 2 (2.33%) | <.05 |
|  | **LVEF (%)** | **Reduced to mildly reduced EF (<50)** | 3 | −0.03 | 0.02 | 0.01 | −0.07 | 0 (0.0%) |  |
|  |  | **Preserved EF (≥50)** | 148 | 0.02 | 0.05 | 0.12 | −0.08 | 8 (5.41%) | >.05 |
|  | **LA diameter (mm)** | **Normal (≤40)** | 82 | 0.02 | 0.05 | 0.12 | −0.08 | 7 (8.54%) |  |
|  |  | **Enlarged (>40)** | 67 | 0.01 | 0.05 | 0.11 | −0.09 | 2 (2.99%) | >.05 |
|  | **LAVI (ml/m^2^)** | **Normal (≤34)** | 84 | 0.02 | 0.05 | 0.12 | −0.08 | 4 (4.76%) |  |
|  |  | **Enlarged (>34)** | 60 | 0.02 | 0.05 | 0.12 | −0.08 | 3 (5.0%) | >.05 |
| **QRS amplitude** | **Age** | **<65** | 116 | 0.08 | 0.53 | 1.12 | −0.96 | 4 (3.45%) |  |
|  |  | **≥65** | 57 | −0.21 | 0.49 | 0.75 | −1.17 | 2 (3.51%) | <.05 |
|  | **Sex** | **Male** | 121 | −0.04 | 0.54 | 1.02 | −1.10 | 4 (3.31%) |  |
|  |  | **Female** | 52 | 0.04 | 0.52 | 1.06 | −0.98 | 1 (1.92%) | >.05 |
|  | **Weight (kg)** | **<70** | 83 | 0.15 | 0.53 | 1.19 | −0.89 | 3 (3.61%) |  |
|  |  | **≥70** | 89 | −0.18 | 0.49 | 0.78 | −1.14 | 5 (5.62%) | <.05 |
|  | **BSA (m^2^)** | **Low BSA (<1.6)** | 25 | 0.20 | 0.48 | 1.14 | −0.74 | 0 (0.0%) |  |
|  |  | **Normal BSA (1.6**–**2.0)** | 119 | −0.01 | 0.55 | 1.07 | −1.09 | 4 (3.36%) |  |
|  |  | **High BSA (>2.0)** | 28 | −0.28 | 0.38 | 0.46 | −1.02 | 1 (3.57%) | <.05 |
|  | **BMI (kg/m^2^)** | **Underweight to normal (<25.0)** | 81 | 0.25 | 0.51 | 1.25 | −0.75 | 2 (2.47%) |  |
|  |  | **Overweight to obese (≥25.0)** | 91 | −0.26 | 0.43 | 0.58 | −1.10 | 7 (7.69%) | <.05 |
|  | **LVEF (%)** | **Reduced to mildly reduced EF (<50)** | 3 | −0.52 | 0.33 | 0.13 | −1.17 | 0 (0.0%) |  |
|  |  | **Preserved EF (≥50)** | 153 | −0.05 | 0.52 | 0.97 | −1.07 | 7 (4.58%) | >.05 |
|  | **LA diameter (mm)** | **Normal (≤40)** | 83 | 0.10 | 0.50 | 1.08 | −0.88 | 2 (2.41%) |  |
|  |  | **Enlarged (>40)** | 71 | −0.27 | 0.42 | 0.55 | −1.09 | 3 (4.23%) | <.05 |
|  | **LAVI (ml/m^2^)** | **Normal (≤34)** | 84 | 0.05 | 0.51 | 1.05 | −0.95 | 3 (3.57%) |  |
|  |  | **Enlarged (>34)** | 65 | −0.21 | 0.49 | 0.75 | −1.17 | 2 (3.08%) | <.05 |
| **T amplitude** | **Age** | **<65** | 116 | −0.04 | 0.12 | 0.20 | −0.28 | 10 (8.62%) |  |
|  |  | **≥65** | 57 | −0.07 | 0.13 | 0.18 | −0.32 | 4 (7.02%) | >.05 |
|  | **Sex** | **Male** | 121 | −0.05 | 0.14 | 0.22 | −0.32 | 8 (6.61%) |  |
|  |  | **Female** | 52 | −0.05 | 0.09 | 0.13 | −0.23 | 2 (3.85%) | >.05 |
|  | **Weight (kg)** | **<70** | 83 | −0.01 | 0.11 | 0.21 | −0.23 | 6 (7.23%) |  |
|  |  | **≥70** | 89 | −0.08 | 0.12 | 0.16 | −0.32 | 7 (7.87%) | <.05 |
|  | **BSA (m^2^)** | **Low BSA (<1.6)** | 25 | −0.03 | 0.09 | 0.15 | −0.21 | 1 (4.0%) |  |
|  |  | **Normal BSA (1.6**–**2.0)** | 119 | −0.04 | 0.13 | 0.21 | −0.29 | 10 (8.4%) |  |
|  |  | **High BSA (>2.0)** | 28 | −0.10 | 0.08 | 0.06 | −0.26 | 3 (10.71%) | <.05 |
|  | **BMI (kg/m^2^)** | **Underweight to normal (<25.0)** | 81 | −0.01 | 0.13 | 0.24 | −0.26 | 6 (7.41%) |  |
|  |  | **Overweight to obese (≥25.0)** | 91 | −0.08 | 0.11 | 0.14 | −0.30 | 6 (6.59%) | <.05 |
|  | **LVEF (%)** | **Reduced to mildly reduced EF (<50)** | 3 | −0.05 | 0.09 | 0.13 | −0.23 | 0 (0.0%) |  |
|  |  | **Preserved EF (≥50)** | 153 | −0.06 | 0.12 | 0.18 | −0.30 | 10 (6.54%) | >.05 |
|  | **LA diameter (mm)** | **Normal (≤40)** | 83 | −0.03 | 0.12 | 0.21 | −0.27 | 5 (6.02%) |  |
|  |  | **Enlarged (>40)** | 71 | −0.11 | 0.11 | 0.11 | −0.33 | 5 (7.04%) | <.05 |
|  | **LAVI (ml/m^2^)** | **Normal (≤34)** | 84 | −0.05 | 0.13 | 0.20 | −0.30 | 5 (5.95%) |  |
|  |  | **Enlarged (>34)** | 65 | −0.09 | 0.11 | 0.13 | −0.31 | 5 (7.69%) | <.05 |

For subgroup analyses, only the last acquired ECG pair (12-lead vs. 6-lead or 12-lead vs. single-lead) from each patient's first visit was utilized. This specific data selection strategy was adopted to ensure that the analysis primarily reflected the influence of individual patient characteristics on ECG parameters, rather than potential variations arising from repeated measurements across multiple visits or within a single visit. Furthermore, participants were included in a specific subgroup analysis only if they had valid and complete data for the respective subgroup variable. For comparisons of numerical ECG parameters (mean differences from Bland-Altman analysis) between subgroups, independent t-tests or Mann-Whitney U tests were used for two subgroups and one-way analysis of variance (ANOVA) or Kruskal-Wallis tests were used for three subgroups. A p-value < 0.05 was considered statistically significant. Subgroup analyses were exploratory and p-values were not adjusted for multiple comparisons.

ECG, electrocardiogram; 12L, 12-lead electrocardiogram; 6L, 6-lead handheld electrocardiogram; 1L, single-lead smartwatch electrocardiogram; BSA, body surface area; BMI, body mass index; LVEF, left ventricular ejection fraction; LA diameter, left atrial diameter; LAVI, left atrial volume index

## Supplementary Figure S1. Representative ECG reports from consumer-grade devices.


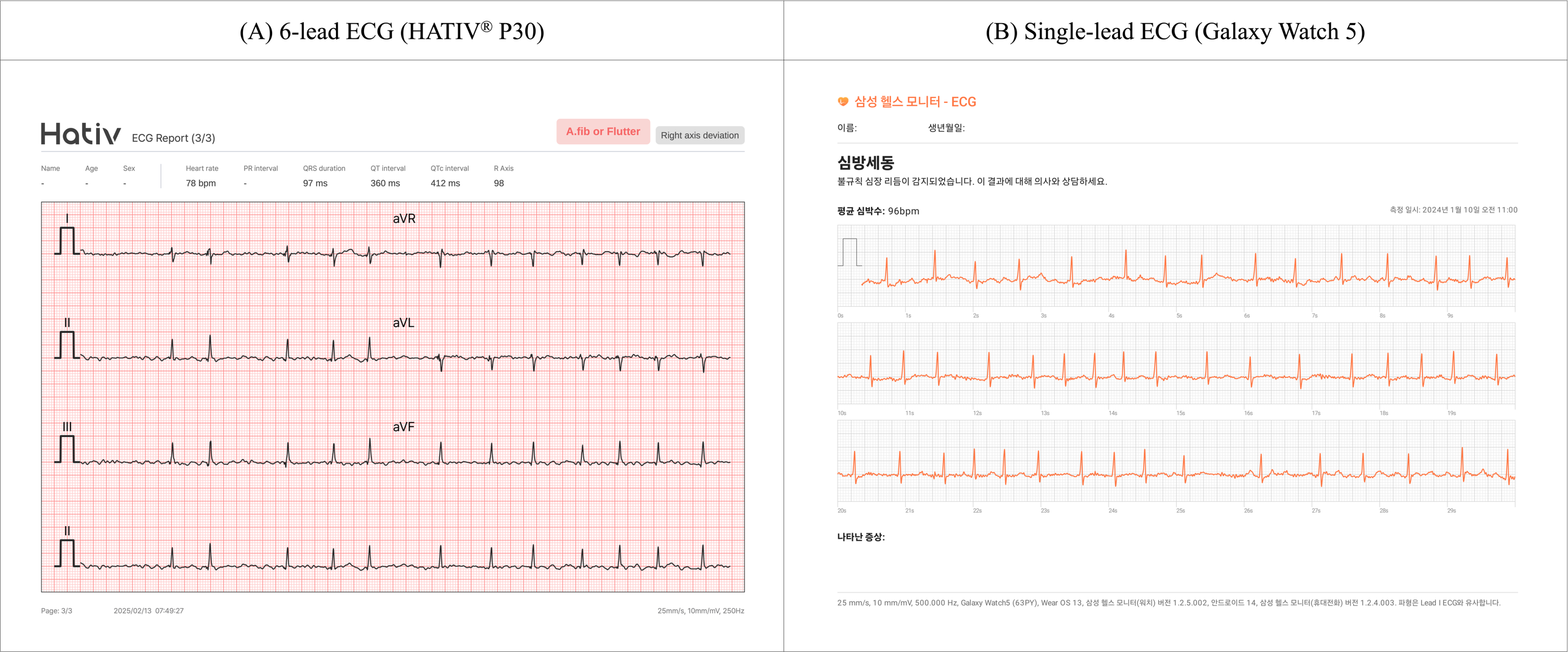


The HATIV device provides a 6-lead ECG report including automated measurements (heart rate, PR, QRS, QT, QTc, and axis) and rhythm interpretation. The Galaxy generates a single-lead ECG report in Korean, which in this case indicates detection of atrial fibrillation (“심방세동”). The HATIV report records the full 30-second ECG; however, for illustrative purposes only a 10-second segment is displayed here.

## Supplementary Figure S2. Representative example of rhythm discordance due to acquisition time gap.

| **12 L** | 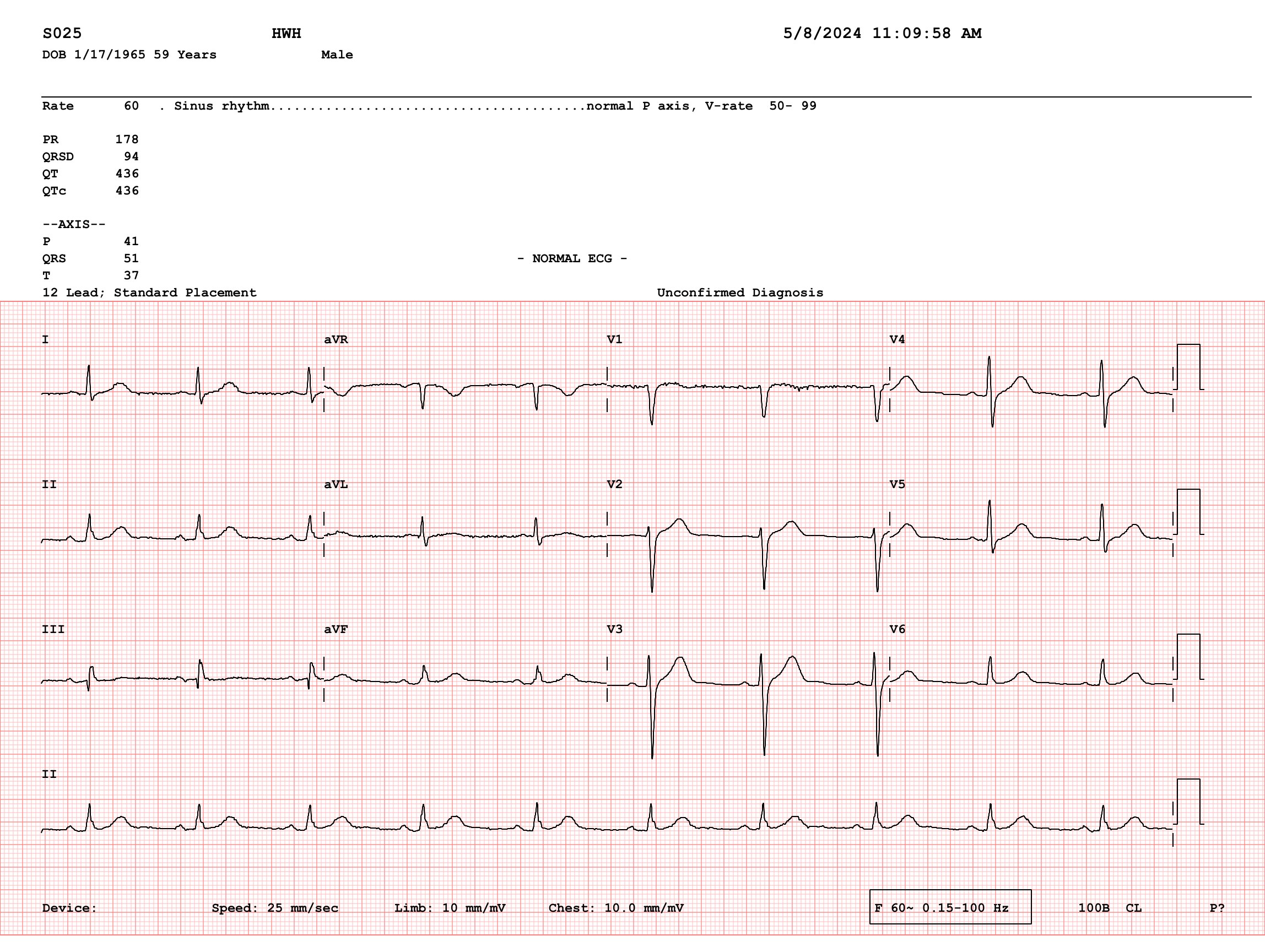 |
| --- | --- |
| **6 L** | 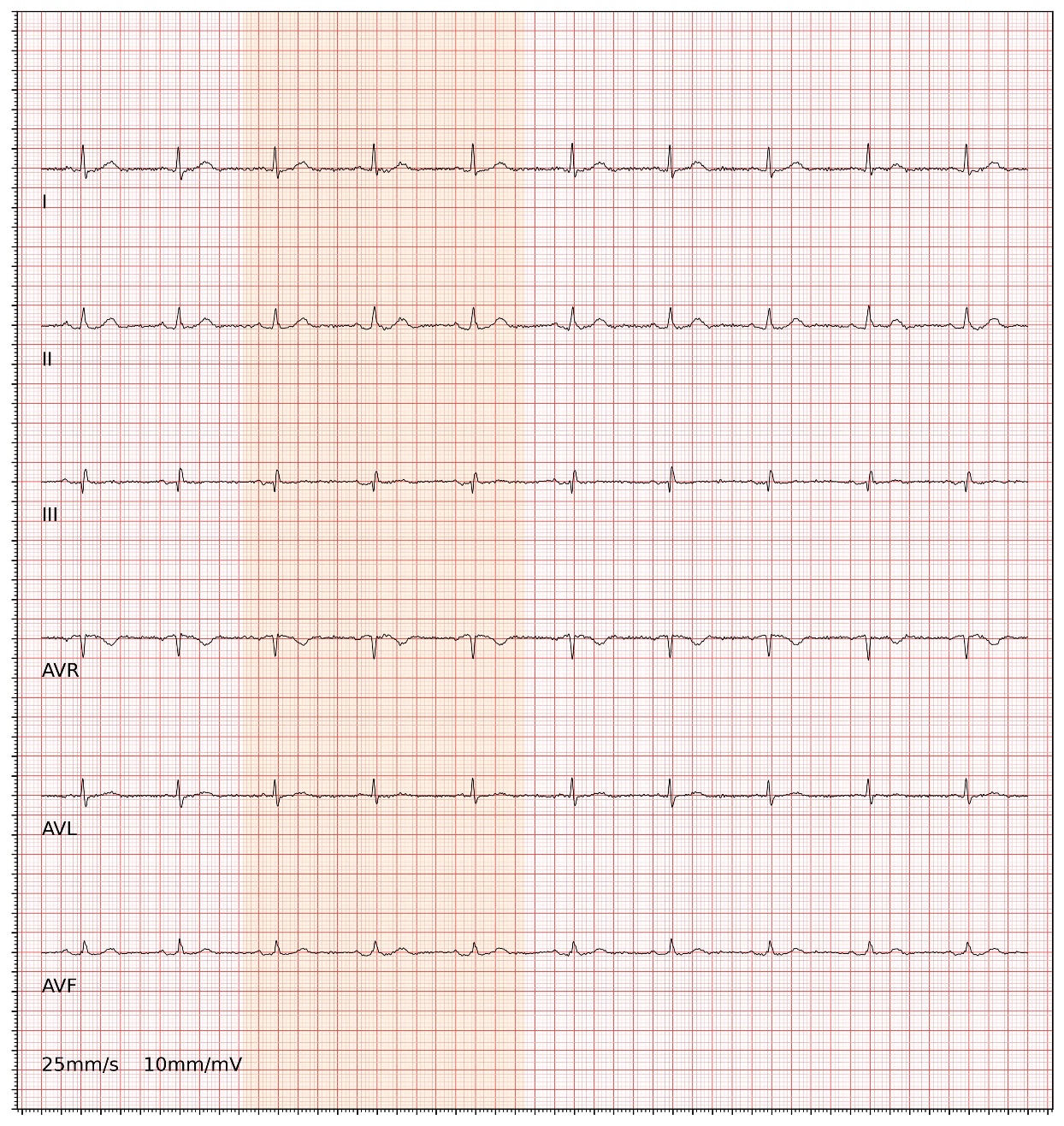 |
| **1 L** | 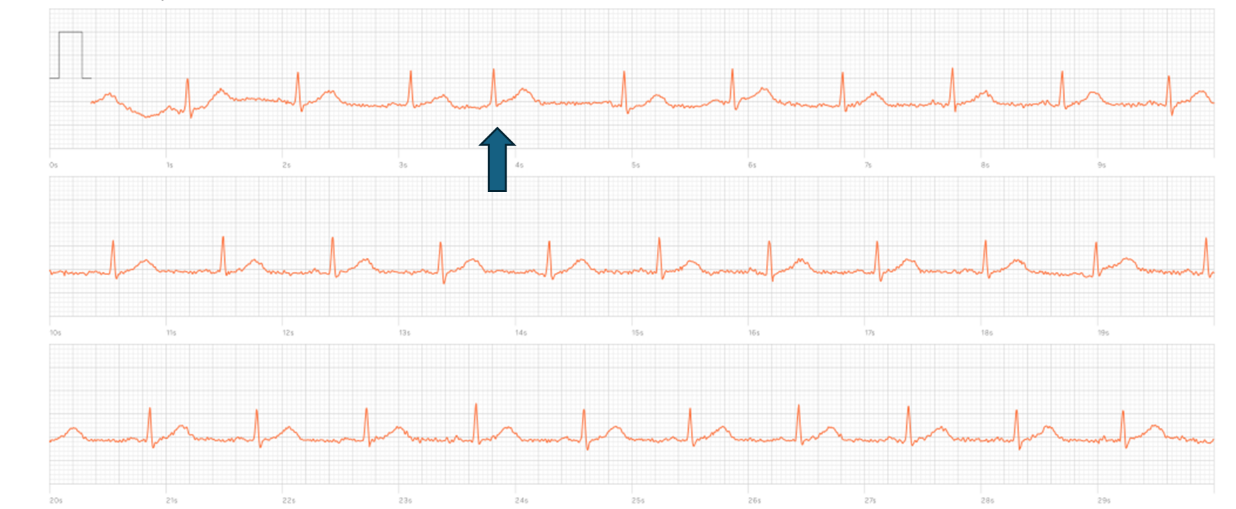 |

Representative example of rhythm discordance attributable to acquisition time gap between simultaneous 12-lead and 6-lead recordings and subsequent sequential single-lead recording. In this case, the simultaneous 12-lead and 6-lead recordings demonstrate sinus rhythm, whereas the later single-lead recording captures transient atrial premature complex (4th beat). This example illustrates the timing-related variability of ectopic beats rather than device misclassification.

12L, 12-lead electrocardiogram; 6L, 6-lead handheld electrocardiogram; 1L, single-lead smartwatch electrocardiogram; APC, atrial premature complex

## Supplementary Figure S3. Bland-Altman plots comparing ECG parameters between 12-lead and consumer-grade dvice ECGs (patient-level analysis).


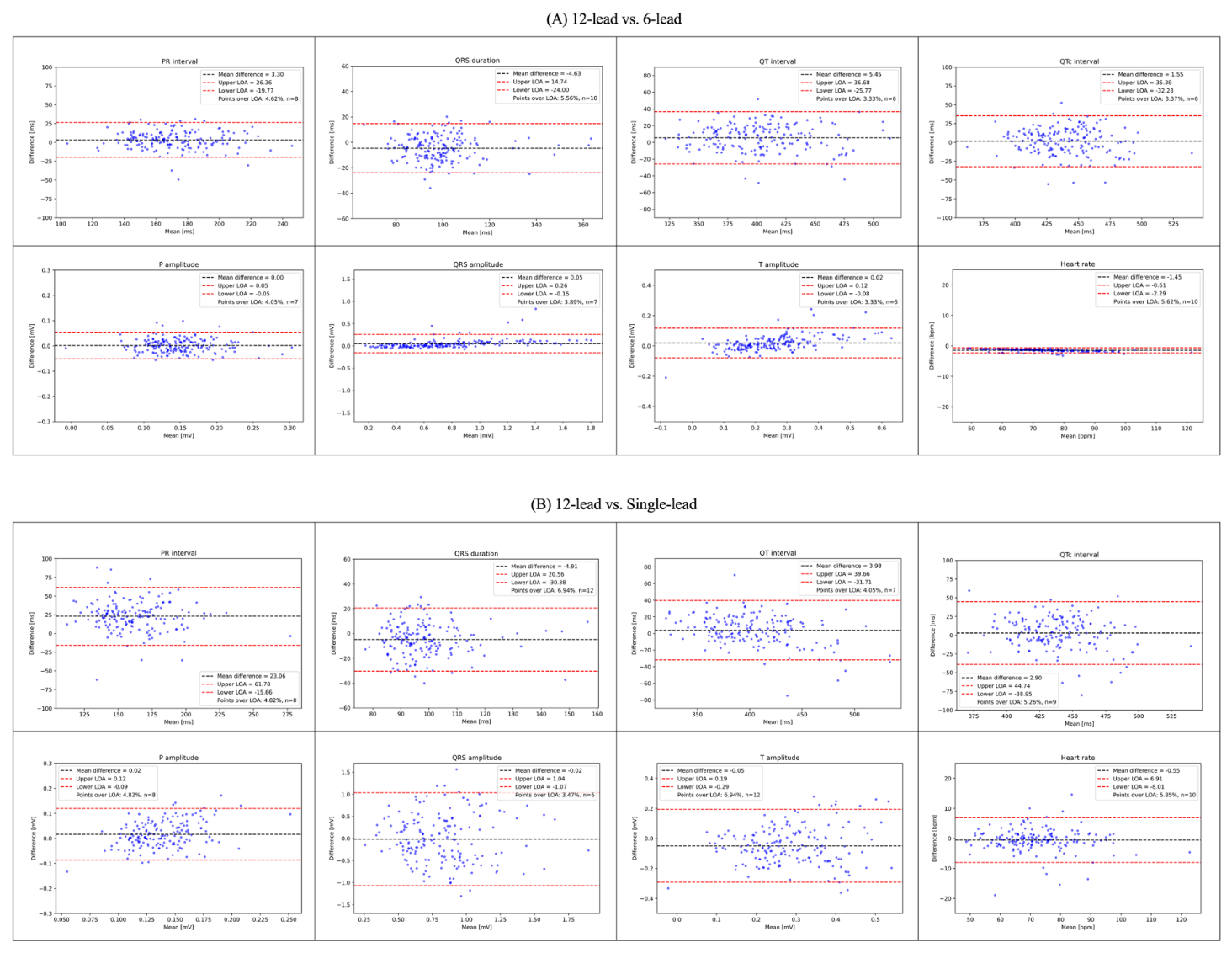

Supplement: ztag086_Supplementary_Data [file ztag086_supplementary_data.docx]
